# A snapshot of the microbiome of blood and ticks of captive cheetahs (*Acinonyx jubatus*) from selected conservation facilities in South Africa

**DOI:** 10.3389/fmicb.2026.1882746

**Published:** 2026-07-20

**Authors:** Kedibone Masenya, Maphuti Betty Ledwaba, Nosipho Khumalo, Sekgota Marcus Makgabo, Sankwetea Prudent Mokgokong, Mamohale Chaisi

**Affiliations:** 1 Department of Botany and Plant Biotechnology, University of Johannesburg, Johannesburg, South Africa; 2 Department of Agriculture and Animal Health, University of South Africa, Roodepoort, South Africa; 3 Foundational Biodiversity Science, SANBI National Zoological Garden, Pretoria, South Africa; 4 Vaccine and Diagnostic Development Programme, Agricultural Research Council, Onderstepoort Veterinary Research, Onderstepoort, South Africa; 5 Department of Veterinary Tropical Diseases, Faculty of Veterinary Science, University of Pretoria, Onderstepoort, South Africa; 6 Unit of Environmental Sciences and Management, North-West University, Potchefstroom, South Africa

**Keywords:** 16S rRNA gene, ASVS, cheetah, conservation, metabarcoding

## Abstract

Cheetahs (*Acinonyx jubatus*) are listed as vulnerable on the IUCN Red List, with populations declining across their native range due to anthropogenic pressures. To support conservation, breeding programs have been established in South Africa and globally. However, captivity introduces new ecological challenges, including increased exposure to ticks and tick-borne pathogens (TBPs). Translocation of cheetahs may further facilitate the spread of pathogens, potentially affecting animal health and introducing infections into new environments. Despite these risks, little is known about the blood and tick microbial communities of captive and free-ranging cheetahs in South Africa. This study investigated the composition, abundance, taxonomic classification, of bacteria detected in captive cheetahs and associated ticks in South Africa. Full-length 16S rRNA gene sequencing was performed on samples originating from 10 adult cheetahs and 20 tick specimens representing 8 tick species from the genera *Amblyomma*, *Haemaphysalis*, *Hyalomma*, and *Rhipicephalus* across five provinces of South Africa. Sequencing was conducted using the PacBio platform, and reads were classified to genus level using the SILVA microbial database at a 99% confidence threshold. Amplicon Sequence Variant (ASV) analysis identified both known and unknown bacterial taxa, including genera harboring potential zoonotic pathogens. Proteobacteria was the dominating bacterial phylum in both the host and tick microbiota, however ticks had a larger proportion of Proteobacteria than hosts. At the genus level, the tick bacterial microbiomes were dominated by the genus *Coxiella*, while host microbiomes were dominated by *Bacillus* and *Stenotrophomonas*. The compositions of dominant genera in female ticks constituted a higher abundance of *Coxiella* and *Methylobacterium-Methylorubrum* compared to males. Accounting for the low-biomass nature and contamination risks of blood, resulted in the Shannon and Simpson diversity indices being significantly higher for host samples, while ticks maintained significantly higher observed ASV richness. Beta-diversity analysis further revealed significant differences in microbial community structure between hosts and ticks (*p* < 0.05), making sample type to be the primary factor shaping bacterial composition. In contrast, sex, province, and tick species did not significantly influence Beta-diversity. Overall, these findings highlight distinct bacterial community patterns between hosts and ticks and emphasize the importance of sample type in structuring tick-associated microbiomes and the importance of continuous surveillance and monitoring of TBPs during wildlife translocation to reduce risks to wildlife, livestock, and human health.

## Introduction

The cheetah (*Acinonyx jubatus*, Schreber, 1775) is found throughout southern Africa (Skinner and Chimimba, 2005; Durant et al., 2017). It is highly threatened, with its range decreasing significantly due to various human-related and ecological threats, including habitat destruction, illegal trade, human-wildlife conflict, and disease (Jeo et al., 2018; Dickman et al., 2018; Naude et al., 2020). As a result, it is listed as vulnerable on the International Union for Conservation of Nature (IUCN) Red List of Threatened Species (Durant et al., 2024). In South Africa, free-ranging cheetahs inhabit open plains in the savannah, along the Limpopo River valley, and are also present in large, protected areas such as the Kruger National Park (Horak et al., 2018). Cheetahs are also bred in captivity, with an estimated population of over 600 individuals across more than 70 facilities in South Africa (Marnewick et al., 2007). They are also maintained as a managed metapopulation across fenced reserves in various provinces, including the Western Cape, Eastern Cape, Gauteng, and Free State through the Metapopulation Initiative (Weise et al., 2015; Magliolo et al., 2023). Captive cheetahs are susceptible to various ailments such as gastrointestinal distress, metabolic disease, reduced reproductive fitness, and bacterial and viral infections (Terio et al., 2004).

The 16S rRNA metabarcoding analysis has emerged as a powerful non-invasive technique for monitoring the health and physiological condition of wild animals, particularly in threatened species that are difficult to access and for which traditional diagnostic tools may be limited or unavailable (Pannoni et al., 2022). Therefore, captive settings provide an opportunity to investigate how diet, disease, or stress may influence microbial profiles. Changes in the host’s microbial composition can signal shifts in immune function, nutritional status, stress, or infection (Potgieter et al., 2015; Trevelline et al., 2019; West et al., 2019). For example, an increased abundance of Proteobacteria in the microbiome has been linked to inflammation and stress-related dysbiosis in both humans and wildlife (Potgieter et al., 2015; Castillo et al., 2019), while the presence or absence of specific bacteria may indicate skin health, wound healing, or exposure to human-influenced environments (Amato et al., 2013). In carnivores, high levels of *Clostridium* spp. or reduced microbial diversity have been associated with dietary changes and may signal gastrointestinal complications (Wasimuddin et al., 2017). Incorporating microbial markers into health assessments could enable early detection of diseases or stress, thereby supporting conservation strategies for threatened species. Additionally, microbial surveillance provides a comprehensive overview of the host microbiome and aids in identifying emerging pathogens and infectious diseases that may threaten the conservation of wildlife (de Cock et al., 2022).

Studies on the blood microbial communities in wild and domestic animals are increasing and highlight the role of blood microbiomes in animal health and welfare, and bio-surveillance (Khoza et al., 2024). Such studies have enhanced our understanding of potential reservoir hosts of emerging pathogens, including vector-borne infections, and changes in the composition and diversity of host microbial communities under different environmental conditions and species management regimes (Amato et al., 2013; Menke et al., 2015; Maly et al., 2024; Kolo et al., 2025; Ontiveros-Chacón et al., 2025; Nyamota et al., 2025). Although over 10 ixodid tick species (Horak et al., 2018; Ledwaba et al., 2022) and tick-borne protozoa have been reported from free-ranging and captive cheetahs in South Africa, there is no information on the microbial communities of cheetahs and associated tick vectors. Previous studies mainly focused on the gut and fecal microbiome of cheetahs (Wasimuddin et al., 2017; Maly et al., 2024) and other felids (Janeczko et al., 2008; Honneffer et al., 2014). Additionally, ticks harbor diverse pathogens and endosymbionts, and their microbial communities may vary according to tick species, stage, sex, and geographical location (Al Masri et al., 2025). Recently, two fatal cases of periorbital damage due to tick infestations in cheetahs, resulting in traumatic myiasis, were reported in cheetahs that were reintroduced to Mozambique from South Africa (Kendon et al., 2025), highlighting the importance of pre- and post-translocation bio-surveillance.

This study provides a snapshot of the bacterial composition, diversity, and abundance, and potential blood-borne pathogens, of captive cheetahs and their ticks using high-throughput sequencing of the 16S rRNA gene. To our knowledge, this is the first report of the microbiome of captive cheetahs in South Africa. It highlights the potential application of microbial surveillance and profiling in health assessments of cheetahs and other threatened wildlife to evaluate the impacts of captivity, translocation, and environmental changes on their health and welfare, thereby informing conservation strategies.

## Materials and methods

### Ethical approval and sample collection

Ethical approval for the study was obtained from the South African National Biodiversity Institute (SANBI) Animal Research Ethics and Scientific Committee (AREC), Project number: SANBI/RES/P2020-15. Permission to carry out research under Section 20 of the Animal Diseases Act, 1984 (Act No. 35 of 1984) was obtained from the Department of Agriculture, Land Reform and Rural Development (DALRRD) (permit no. SDAH-Epi-21032313211). Additionally, permission for sampling was obtained from the participating game reserves and conservation facilities.

Twenty-four whole blood samples and 22 ticks were collected opportunistically from cheetahs at seven conservation facilities in South Africa during routine health surveys and/or translocations, as part of the Metapopulation Initiative (TMI) project and other initiatives. The blood samples and ticks analyzed in this study were collected as part of a wider study on ticks and tick-borne pathogens of felids in South Africa. Blood samples were collected into sterile EDTA vacutainer tubes and stored at 4 °C at the SANBI Wildlife Biobank until further analysis. Ticks were collected in airtight containers containing 70% ethanol for preservation.

Of the 22 ticks collected, only 20 (three nymphs, nine females, and eight males) belonging to four genera (*Amblyomma*, *Haemaphysalis*, *Hyalomma*, and *Rhipicephalus*) were included in the analysis, as two specimens did not meet the quality control requirements. Table 1 shows the detailed metadata of the samples.

**TABLE 1:** Blood and tick samples analyzed in this study.

Province	Host information			Tick information		
	Host ID	sex	age	Tick ID species life stage		
Eastern Cape	**FEL32**	Male	Sub-adult	FEL32T	*Amblyomma marmoreum*	Nymph
KwaZulu-Natal	**FEL34**	Male	Sub-adult	FEL34T	*Hyalomma truncatum*	Male
	FEL08	Female	Adult		–	
	FEL09	Male	Adult	FEL9aT	*Amblyomma hebreaum*	Male
				FEL9cT	*Rhipicephalus simus*	Male
				FEL9dT	*Rhipicephalus africanus*	Female
	**FEL36**	Male	Adult	FEL36T	*Haemaphysalis elliptica*	Female
	FEL24	Male	Adult	FEL 24cT	*Amblyomma hebreaum*	Male
	**FEL 25**	Male	Adult	FEL 25bT	*Hyalomma truncatum*	Female
Limpopo	**FEL 26**	Male	Sub-adult	FEL 26aT	*Rhipicephalus zambeziensis*	Male
	**FEL 27**	Female	Sub-adult	FEL 27bT	*Amblyomma hebreaum*	Male
				FEL 27cT	*Amblyomma hebreaum*	Female
	**FEL28**	Male	Adult	FEL 28bT	*Rhipicephalus simus*	Female
	**FEL30**	Female	Sub-adult	FEL 30aT	*Amblyomma marmoreum*	Nymph
				**FEL 30bT**	*Rhipicepalus zambeziensis*	Male
	**FEL 31**	Female	Adult	FEL 31T	*Amblyomma hebreaum*	Male
North West	FEL 11	Male	Sub-adult	NTC^a^	NTC	NTC
	FEL 13	Male	Sub-adult	NTC	NTC	NTC
	FEL 14	Male	Sub-adult	NTC	NTC	NTC
	FEL 15	Male	Sub-adult	NTC	NTC	NTC
	FEL 17	Male	Sub-adult	NTC	NTC	NTC
	**FEL18**	Male	Adult	FEL 18bT	*Haemaphysalis elliptica*	Nymph
	**FEL 19**	Male	Adult	FEL 19T	*Rhipicephalus sulcatus*	Male
	FEL 20	Female	Adult	FEL 20T	*Haemaphysalis elliptica*	Female
	**FEL 21**		Sub-adult	FEL 21aT	*Haemaphysalis zumpti*	Female
				FEL 21bT	*Haemaphysalis elliptica*	Female
	**FEL 22**	Male	Unknown	FEL 22bT-22at	*Haemaphysalis elliptica*	Female
Western Cape	FEL 33	Female	Sub-adult	–		
	FEL 34	Male	Sub-adult	FEL34T	*Hyalomma truncatum*	Male

NTC^a^, No ticks collected; Samples in bold did not meet the required QC and were not sequenced.

### Morphological and molecular identification of ticks

Ticks were identified to species level by a taxonomist (Dr Deon Bakkes) at the Getrud Theiler Tick Museum using a Zeiss Discovery V20 Stereomicroscope (Zeiss Research Microscopy Solutions, Zeiss Group) and published taxonomic keys (Banks, 1908; Walker et al., 2000; Horak et al., 2018).

Individual adult ticks were surface sterilized by rinsing three times with phosphate-buffered saline (PBS) containing 0.05% Tween 20 (0.05% PBS-Tween) to remove contaminants. Whole ticks were subsequently crushed using a sterile mortar and pestle and incubated at 55 °C for 24 h before DNA extraction. Genomic DNA was extracted using the Zymo Quick-DNA™ Miniprep Kit (for whole blood) and the Quick-DNA™ Insect Miniprep Kit (for ticks) (Zymo Research, Freiburg, Bresgau, Germany), respectively, following the manufacturers’ protocols. Species identification was confirmed by Sanger sequencing and DNA barcoding of the COI and 16S rRNA genes of the ticks using primers HCO2198 and LCO1490 (Folmer et al., 1994) and 16S + 1 and 16S−1 (Black and Piesman, 1994).

### PCR amplification and sequencing

Genomic DNA from both blood and ticks was submitted to Inqaba Biotechnical Industries (Pty) Ltd., (Pretoria, South Africa) for sequencing. The universal primer set 27F (AGRGTTYGATYMTGGCTCAG) and 1492R (RGYTACCTTGTTACGACTT) (Lane, 1991) was used to amplify the full-length 16S rRNA gene from the genomic DNA. The forward and reverse 16S primers were tailed with sample-specific PacBio barcode sequences to allow for multiplexed sequencing. The KAPA HiFi Hot Start DNA Polymerase (KAPA Biosystems) was used to perform 27 cycles of PCR amplification, with denaturing at 95 °C for 30 s, annealing at 57 °C for 30 s and extension at 72 °C for 60 s. The quality control post-amplification was performed using the Qubit HS DNA kit (Qubit Fluorometer, Invitrogen, USA) and Fragment Analyzer (Agilent Technologies, USA). The amplified DNA from each sample was pooled in equimolar concentration before library preparation. Amplified DNA pool was processed with the SMRTbell Express Template Prep Kit 2.0 (PacBio, USA). To create highly accurate readings (>QV40), raw subreads were processed using the SMRTlink (v7.0.1) circular consensus sequences (CCS) method. Pacific Bioscience data were demultiplexed, and CCS were called using the SMRT-Link analysis software (v9).

### Bioinformatics analysis

Data analysis was done using R v.3.5.1 and Bioconductor v.3.0. R software package DADA2 on HIFI reads (Callahan et al., 2016). Briefly, the sequences were reoriented, and forward and reverse primers were filtered and trimmed according to sequence length and average quality. PacBio error model was used to dereplicate and estimates sequencing errors and to infer the compositions of the samples. Amplicon sequence variants (ASVs) identified as chimeric were removed, and the rest of the ASVs were annotated against the SILVA v138.1 database using the naive Bayesian classifier method from DADA2 (Quast et al., 2013).

### Contaminant identification and *in silico* decontamination

Decontamination was performed using the R Decontam R package (v1.32.0) to account for possible background DNA contamination introduced during sample collection, DNA extraction or library preparation (Davis et al., 2018). The prevalence-based contaminant identification model (method = “prevalence”) was applied due to the low-throughput nature of the negative control cohort. The prevalence of observed sequences was compared in real biological samples to dedicated negative control samples, extracted from laboratory-grade molecular water (Thermo Fisher Scientific, Waltham, MA, USA), and collection buffer controls (Wash buffer). The probability that an ASV is a contaminant was modeled based on its cross-cohort prevalence. The classification threshold for a contaminant ASV was set at the very strict level of 0.5. This is defined such that, if the probability of occurrence of the ASV in the negative control sample was higher than in the biological sample, the ASV was classified as a contaminant. These contaminating ASVs were filtered out of the sequence feature matrix for all samples. After the removal of contaminating sequences, all other biological sequences were kept, except for the negative control samples, which were discarded.

Graphical taxa visualizations were performed in R v.3.5.1 and Bioconductor v.3.0 with phyloseq, ggplot2, vegan, and dplyr R packages (McMurdie and Holmes, 2013). Taxonomic composition was summarized at the phylum and genus level and visualized using relative abundance barplots, based on proportional read counts per sample. The samples were grouped according to sex, province, sample type, and tick species. Alpha diversity was assessed using Shannon, observed richness, and Simpson indices. Differences between groups were tested using Kruskal-Wallis tests, with results visualized using boxplots. Beta diversity was evaluated using Bray-Curtis dissimilarity. Community structure was visualized using Principal Coordinates Analysis (PCoA). Differences in community composition between groups were tested using PERMANOVA with 999 permutations. The statistical significance was considered at *p* < 0.05.

## Results

### Number of reads per sample

Sequencing depth varied substantially across the dataset, with read counts ranging from 332 to 71,176 reads per sample. A total of 356,876 reads were generated across all the samples, with an average sequencing depth of 11,152 reads and a median depth of approximately 4,779 reads per sample. The distribution was highly uneven, with several samples sequenced deeply (>20,000 reads), including FEL-30aT (71,176 reads), FEL-36T (30,565 reads), FEL-27cT (30,012 reads), and FEL-25bT (28,466 reads), while 4 samples had relatively shallow coverage (<1,000 reads), Supplementary Figure 1.

### Decontam results

Contamination analysis was done via a prevalence-based approach. This resulted in very few ASVs being filtered out as contaminants. A high proportion of ASVs was retained as shown in Supplementary Figures 2A, B. The plot of decontam prevalence scores for ASVs indicates two populations, where the first group contains non-contaminants, while the other population consists of probable contaminants as indicated by the 0.5 threshold. Most ASVs were concentrated above the threshold, primarily between ∼0.55 and 0.9, indicating that most sequences are consistently present across samples and are therefore classified as true biological signals. A few ASVs falling below the threshold (0.5), especially those that clustered close to 0, were classified as probable contaminants, Supplementary Figure 2C.

A total of 32 samples were processed. Among these, the read retention rate was consistently high, with a median retention of 99.8% and a mean of 97.2%. While majority of the samples maintained near-perfect recovery, two samples (FEL-26aT-16S and FEL-34T-16S) showed reduced retention rates of 68.9% and 83.0%, respectively, likely reflecting variations in initial sequence quality.

### Retained reads with decontam

To ensure the integrity of the downstream analysis, we evaluated the effect of contaminant removal using the decontam package. A total of 32 samples were processed, including experimental samples and negative controls (e.g., ddH2O.fastq.gz and WashBuffer.fastq.gz).

As shown in Supplementary Figure 3, our filtering pipeline effectively distinguished between biological samples and potential contaminants. While the negative controls and samples with low initial read depth exhibited minimal retention, the experimental samples showed high read retention post-filtering. Among the 20 samples subjected to the full pipeline, the median read retention rate was 99.8% (mean: 97.2%). Two samples (FEL-26aT-16S and FEL-34T-16S) showed moderately reduced retention (68.9% and 83.0%, respectively.

### Rarefaction

Four samples with sequencing depths below 1,000 reads were excluded prior to rarefaction. The remaining samples were rarefied to 1,000 reads per sample to retain sufficient sample representation while minimizing biases associated with uneven sequencing depth. See below the rarefaction plots before and after being rarefied at 1,000 reads (Supplementary Figures 4A, B). These samples were excluded from further processing.

### Comparison of bacterial phylum-level composition between the host and the tick

**FIGURE 1:**
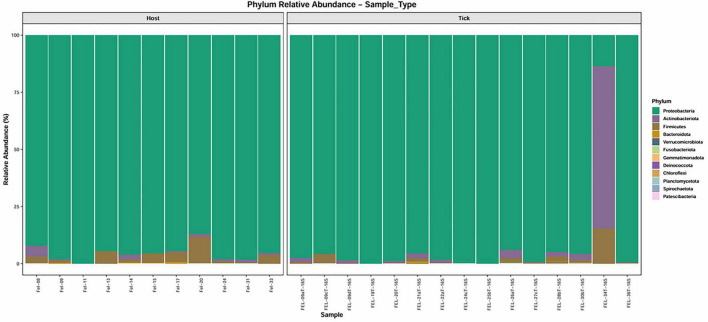
The relative abundance of bacterial phyla between the host and tick.

At the phylum level, Proteobacteria dominated both host and tick microbiomes, however, this was relatively more abundant in ticks (77.22%) compared to hosts (62.62%). In hosts, Firmicutes (25.23%) and Actinobacteriota (10.01%) were the next most abundant phyla after Proteobacteria, while Bacteroidota (1.91%) was found in low abundance. Conversely, tick microbiomes showed an increased abundance of Actinobacteriota (13.31%) along with a decrease in the abundance of Firmicutes (7.42%) and Bacteroidota (1.16%). Additionally, some of the least abundant phyla were found to be unique to ticks and included Verrucomicrobiota (0.34%), Fusobacteriota (0.22%), Deinococcota (0.08%), and Gemmatimonadota (0.07%). In general, both hosts and ticks had distinct microbial communities comprising Proteobacteria, with ticks having more dominant bacterial phyla, Figure 1.

### Comparison of bacterial phylum-level composition between different sexes and life stage

Proteobacteria was the most represented in both male and female tick samples, with a higher relative abundance in female ticks (79.06%) compared to male ticks (70.40%). Following Proteobacteria the most abundant phyla in female ticks were Actinobacteriota (10.41%), Firmicutes (8.62%), Bacteroidota (1.26%), Verrucomicrobiota (0.44%), and Spirochaetota (0.10%). Actinobacteriota was also the second most represented phylum among male ticks, with a significantly greater relative abundance compared to female ticks (19.46%).

**FIGURE 2:**
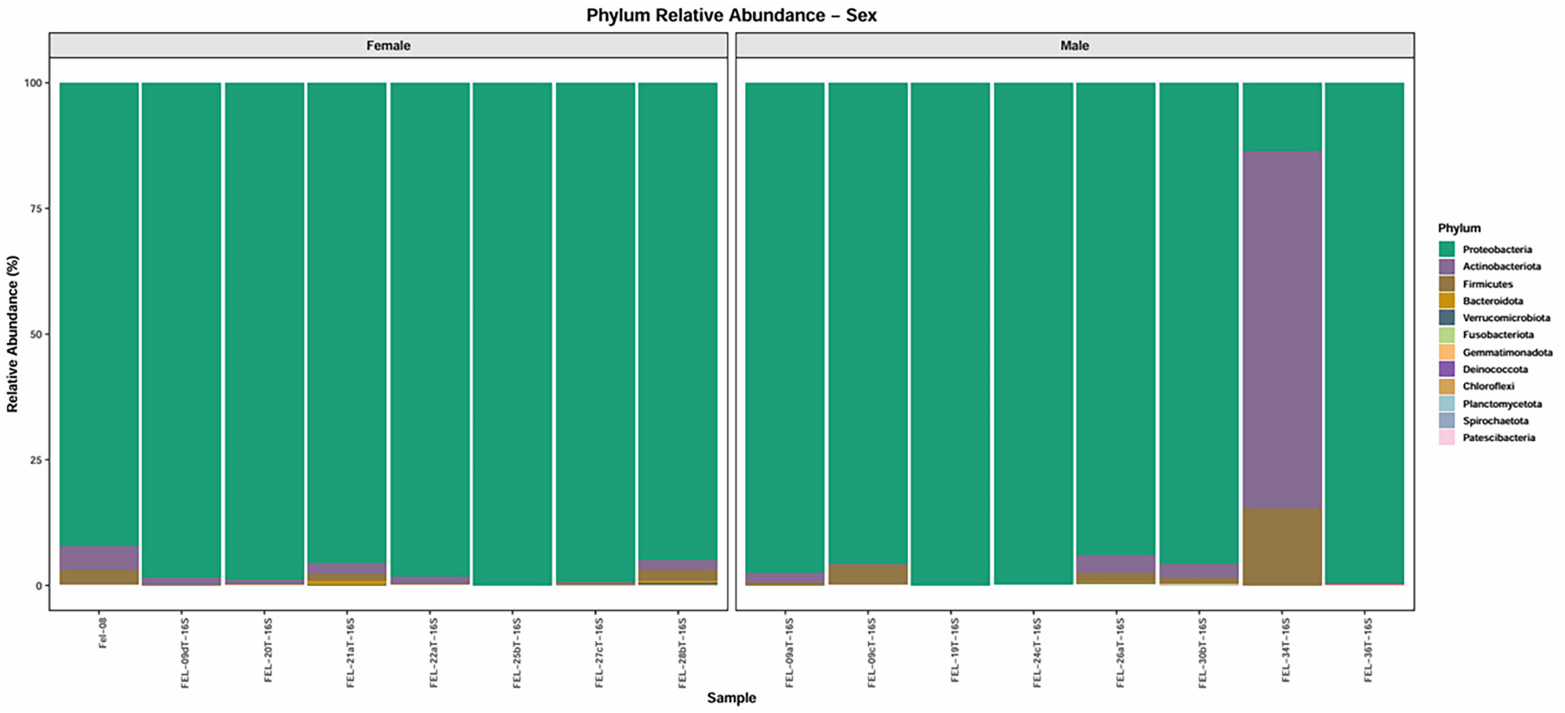
The relative abundance of bacterial phyla across the different sexes.

The low-abundance phyla identified exclusively among males included Fusobacteriota (0.41%), Deinococcota (0.14%) and Planctomycetota (0.10%). Overall, both sexes exhibited broadly similar phylum-level community profiles dominated by Proteobacteria; however, males harbored a higher proportion of Actinobacteriota, Figure 2.

### Comparison of bacterial phylum-level composition across provinces

**FIGURE 3:**
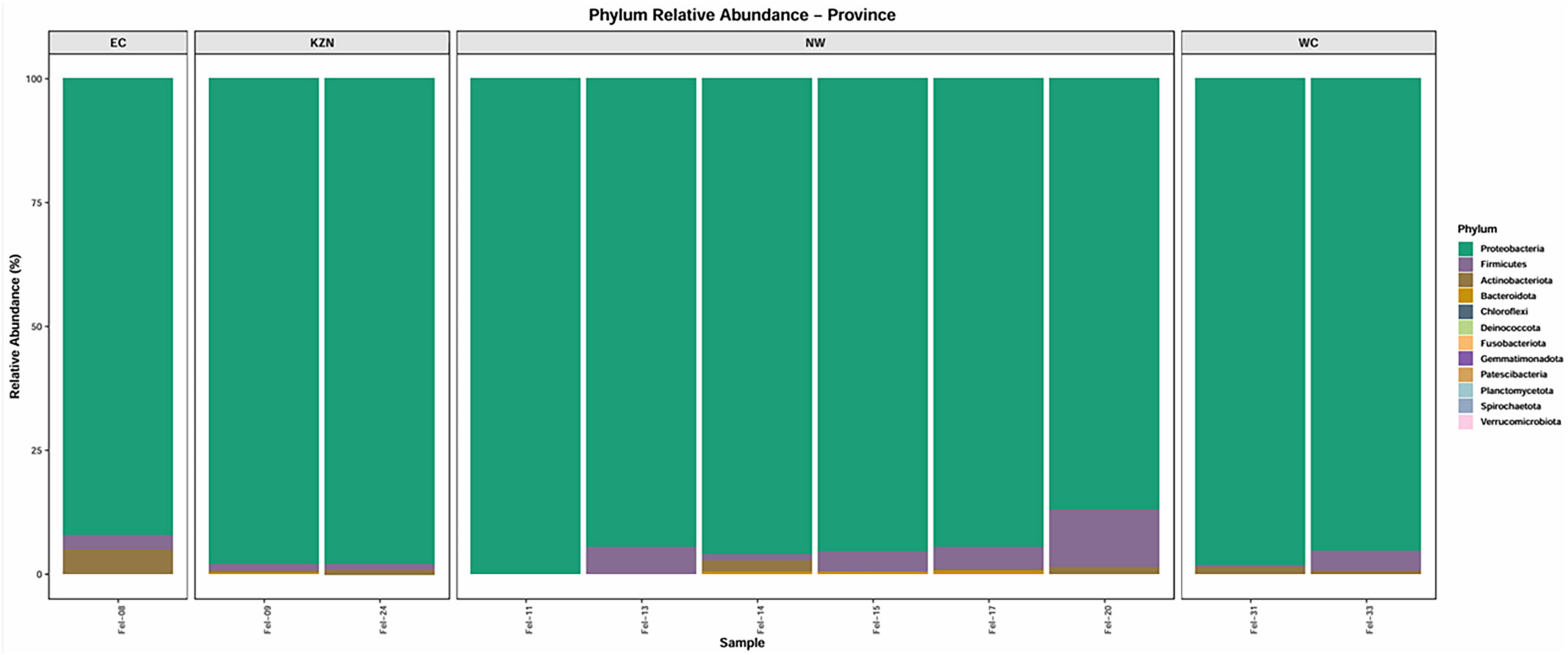
The relative abundance of bacterial phyla across the provinces.

Phylum-based comparison revealed differential community composition among provinces. The Eastern Cape Province was characterized by a high relative abundance of Actinobacteriota (39.25%), Proteobacteria (37.38%), and Firmicutes (23.36%) phyla while, KwaZulu-Natal Province was predominantly represented by Proteobacteria (84.71%). North West and Western Cape Provinces were found to be mostly dominated by Proteobacteria (57.40%, 68.82%, respectively), followed by Firmicutes and Actinobacteriota. Bacteroidota was unique to KwaZulu-Natal and North West provinces and occurred at a very low abundance of <4%, Figure 3.

### Comparison of bacterial phylum-level composition across tick species

**FIGURE 4:**
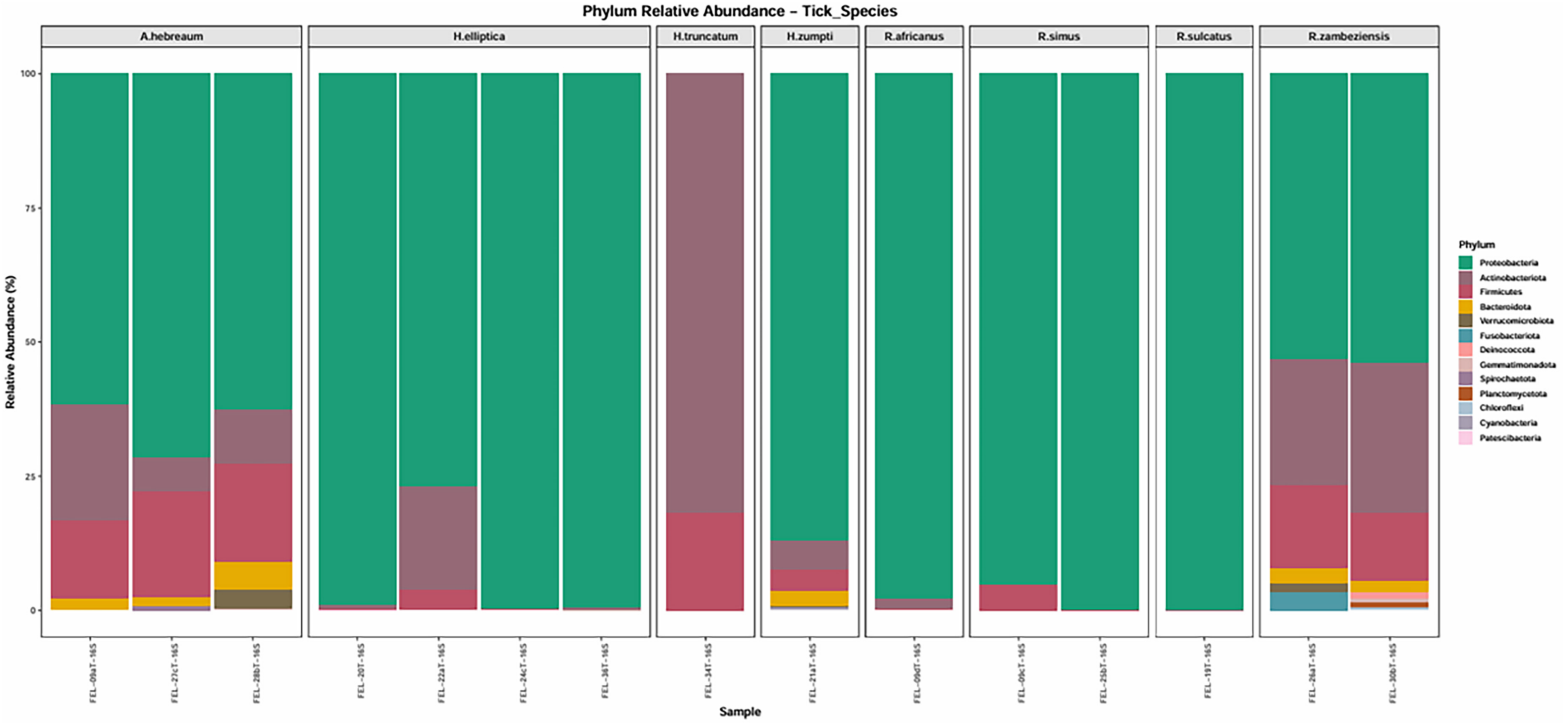
The relative abundance of bacterial phyla across the different tick species.

There was a dominance of phylum Proteobacteria in most of the tick species, accounting for more than 87% of the total sequences in *Ha. elliptica*, *Ha. zumpti*, *R. africanus*, *R. simus*, and *R. sulcatus*. On the contrary, *Hy. truncatum* was dominated by Actinobacteriota making up to 81.83% of the bacterial composition. In contrast, the two tick species *A. hebraeum* and *R. zambeziensis* showed dominance of three different phyla that include Proteobacteria, Firmicutes, and Actinobacteriota. Other minor phyla present included Bacteroidota, Verrucomicrobiota, Fusobacteriota, Deinococcota, Planctomycetota, Chloroflexi and Gemmatimonadota. These were primarily associated with *R. zambeziensis* and *A. hebraeum*. These findings indicate considerable species-specific variation in phylum-level bacterial community composition (Figure 4).

### Comparison of bacterial genus-level composition between hosts and ticks

Based on the relative abundance, *Sphingobium* was identified as the most prevalent genus, representing approximately 44.0% of the entire bacterial group. The next highest represented genera were *Bacillus* (19.0%), *Stenotrophomonas* (8.1%), *Leifsonia* (5.6%), and *Staphylococcus* (4.2%). Other moderately abundant genera comprised *Bradyrhizobium* (2.8%) and *Bifidobacterium* (2.3%). The least abundant genera included *Bartonella* (1.4%), *Massilia* (1.3%), *Methylobacterium-Methylorubrum* (1.1%), *Atopobium* (1.1%), and *Chryseobacterium* (1.0%). While the lowest abundant genera were *Finegoldia* (0.85%), *Erysipelatoclostridium* (0.84%), *Friedmanniella* (0.74%), *Cupriavidus* (0.63%), *Pelomonas* (0.54%), *Novosphingobium* (0.39%), *Hafnia-Obesumbacterium* (0.37%), *Brevundimonas* (0.34%), *Anaplasma* (0.30%), and *Enhydrobacter* (0.29%).

**FIGURE 5:**
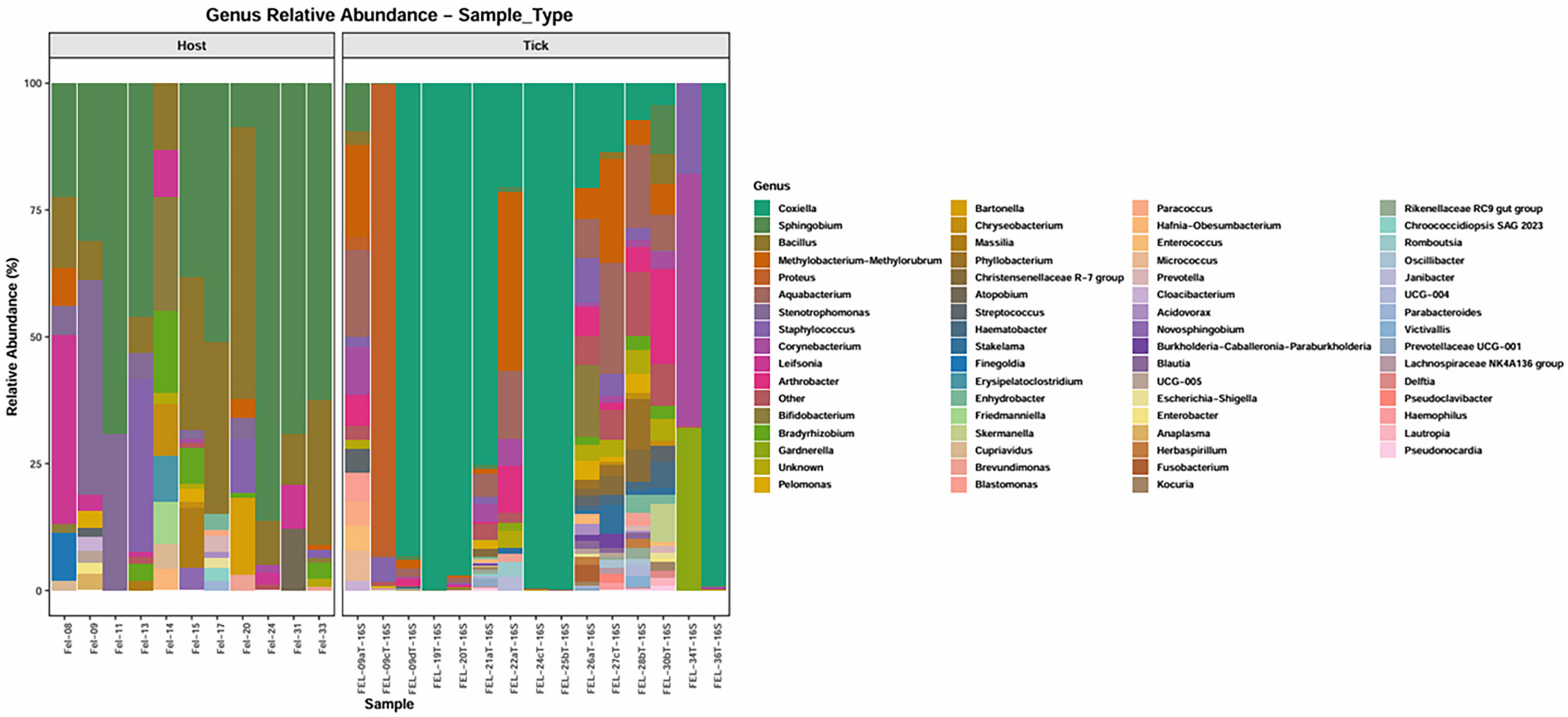
The relative abundance of bacterial genera between ticks and hosts.

Relative abundance analysis of the tick-associated microbiome revealed that *Coxiella* was the dominant genus, comprising approximately 48.7% of the bacterial community. This was followed by *Proteus* (6.4%), *Methylobacterium-Methylorubrum* (6.3%), *Aquabacterium* (6.1%), *Corynebacterium* (5.0%), *Arthrobacter* (3.3%), *Staphylococcus* (2.7%), *Gardnerella* (2.2%), and *Sphingobium* (1.4%). Collectively, these dominant genera constituted the majority of the tick microbiome composition. Genera with relative abundances below 1% collectively accounted for approximately 10.2% of the bacterial community. These included *Bifidobacterium* (0.95%), *Phyllobacterium* (0.92%), Christensenellaceae R-7 group (0.85%), *Bacillus* (0.72%), *Pelomonas* (0.67%), *Streptococcus* (0.65%), *Haematobacter* (0.65%), *Stakelama* (0.64%), *Skermanella* (0.51%), *Bradyrhizobium* (0.47%), *Blastomonas* (0.45%), *Enhydrobacter* (0.40%), *Enterococcus* (0.39%), *Micrococcus* (0.39%), *Paracoccus* (0.36%), *Burkholderia-Caballeronia-Paraburkholderia* (0.27%), *Blautia* (0.27%), *Acidovorax* (0.23%), *Herbaspirillum* (0.22%), *Fusobacterium* (0.22%), *Kocuria* (0.22%), Rikenellaceae RC9 gut group (0.21%), *Chryseobacterium* (0.21%), *Brevundimonas* (0.21%), *Romboutsia* (0.20%), *Oscillibacter* (0.19%), *Janibacter* (0.18%), UCG-004 (0.18%), *Hafnia-Obesumbacterium* (0.15%), *Prevotella* (0.15%), *Victivallis* (0.14%), *Cloacibacterium* (0.14%), Prevotellaceae UCG-001 (0.13%), Lachnospiraceae NK4A136 group (0.12%), UCG-005 (0.12%), *Delftia* (0.12%), *Pseudoclavibacter* (0.11%), *Escherichia-Shigella* (0.11%), *Haemophilus* (0.10%), *Lautropia* (0.10%), *Pseudonocardia* (0.10%), *Enterobacter* (0.09%), *Atopobium* (0.03%), *Chroococcidiopsis* SAG 2023 (0.03%), *Parabacteroides* (0.03%), Other (0.03%), *Cupriavidus* (0.01%), and *Leifsonia* (0.002%), Figure 5.

### Comparison of bacterial genus-level composition across different sexes

**FIGURE 6:**
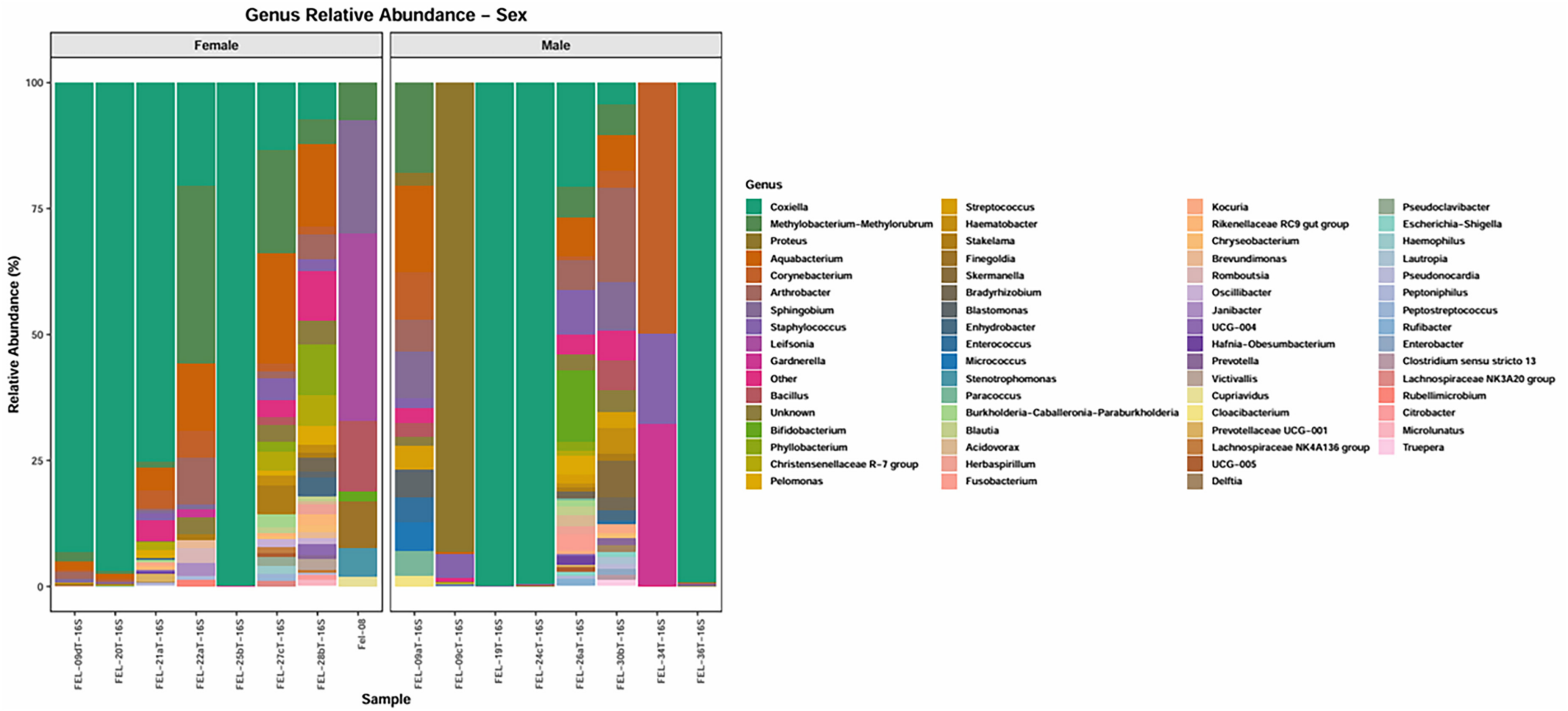
The relative abundance of bacterial genera across different sexes.

The relative abundance analysis in female ticks showed that the most abundant bacterial genus was *Coxiella*, which made up almost 51% of the bacterial community. This was followed by *Methylobacterium-Methylorubrum*, *Aquabacterium*, *Leifsonia*, *Sphingobium*, and *Arthrobacter*, making about 9%, 7%, 5%, 3%, and 2% of the bacteria, respectively. These bacterial genera made up the major portion of the bacterial community present in the female tick. The relative abundance analysis in males showed that the most dominant genus of bacteria was *Coxiella*, which made up about 41% of the bacterial community. This was followed by *Proteus*, *Corynebacterium*, *Staphylococcus*, *Aquabacterium*, and *Gardnerella*, making up about 12%, 8%, 4%, 4%, and 4% respectively, Figure 6.

### Comparison of genus-level composition across different provinces

The relative abundance analysis by province showed different profiles of bacterial communities in the sampled regions. *Leifsonia* was the most abundant genus in the Eastern Cape (EC), which accounted for ∼37.4% of the bacterial community followed by *Sphingobium* (22.4%), *Bacillus* (14.0%), *Finegoldia* (9.3%), *Methylobacterium-Methylorubrum* (7.5%) and *Stenotrophomonas* (5.6%). Lower abundance genera were *Bifidobacterium* and *Cupriavidus* (1.9% each). *Sphingobium* was the most dominant genera in KwaZulu-Natal (KZN) (58.7%) followed by *Stenotrophomonas* (21.1%) and *Bacillus* (8.3%). Other genera were *Leifsonia* (2.9%), *Anaplasma* (1.7%), *Pelomonas* (1.7%), *Cloacibacterium* (1.4%), *Enterobacter* (1.1%) and UCG-005 (1.1%). *Streptococcus* and *Corynebacterium* were found at lower abundances (<1%).

**FIGURE 7:**
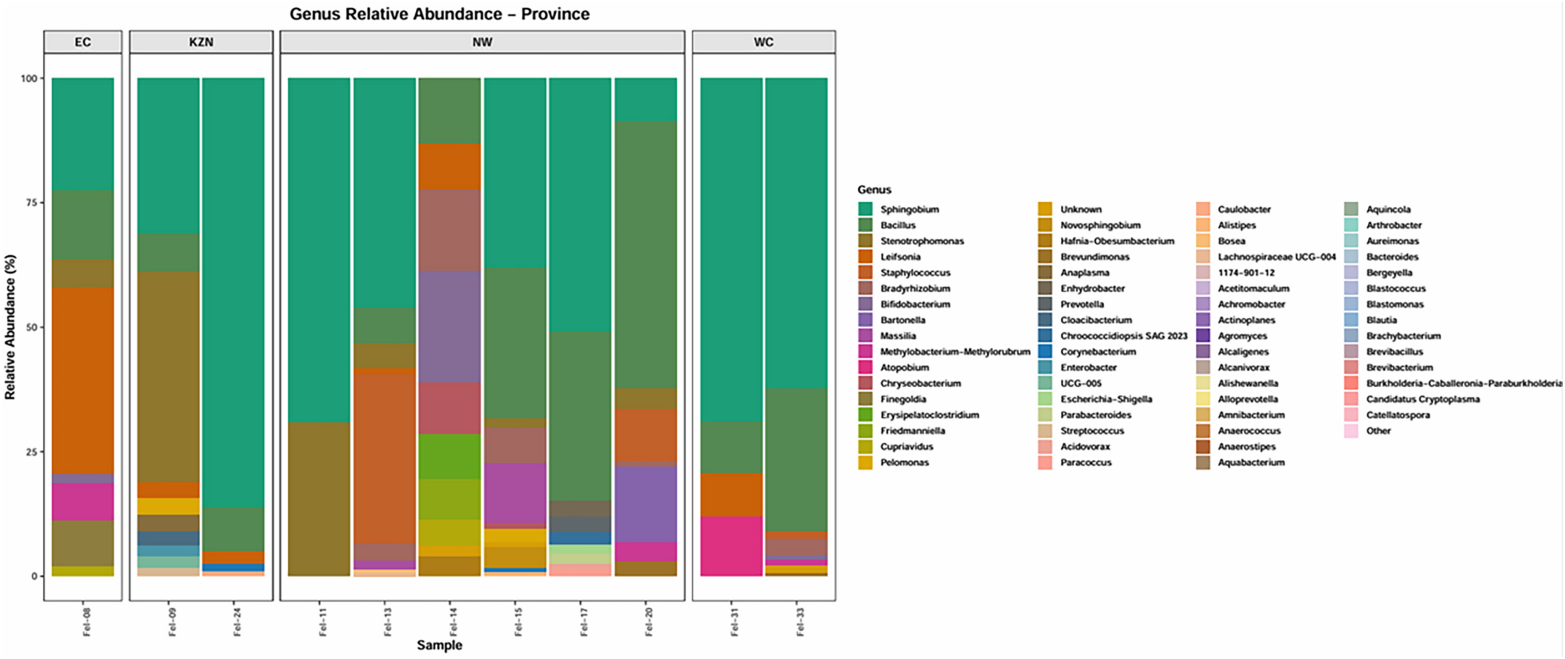
The relative abundance of bacterial genera across different provinces.

The dominant genera in the North West (NW) province were *Sphingobium* (35.5%) and *Bacillus* (23.1%), followed by *Staphylococcus* (7.4%), *Stenotrophomonas* (6.9%), *Bradyrhizobium* (4.7%) and *Bifidobacterium* (3.7%). Other genera included *Bartonella* (2.6%), *Massilia* (2.3%), *Chryseobacterium* (1.9%), *Leifsonia* (1.7%), *Erysipelatoclostridium* (1.5%) and *Friedmanniella* (1.4%). In the Western Cape (WC), the bacterial population distribution included *Sphingobium*, *Bacillus*, *Atopobium*, *Leifsonia*, *Bradyrhizobium*, *Staphylococcus*, *Bifidobacterium*, *Methylobacterium-Methylorubrum*, and *Brevundimonas*, Figure 7.

### Comparison of bacterial genus-level composition across different tick species

**FIGURE 8:**
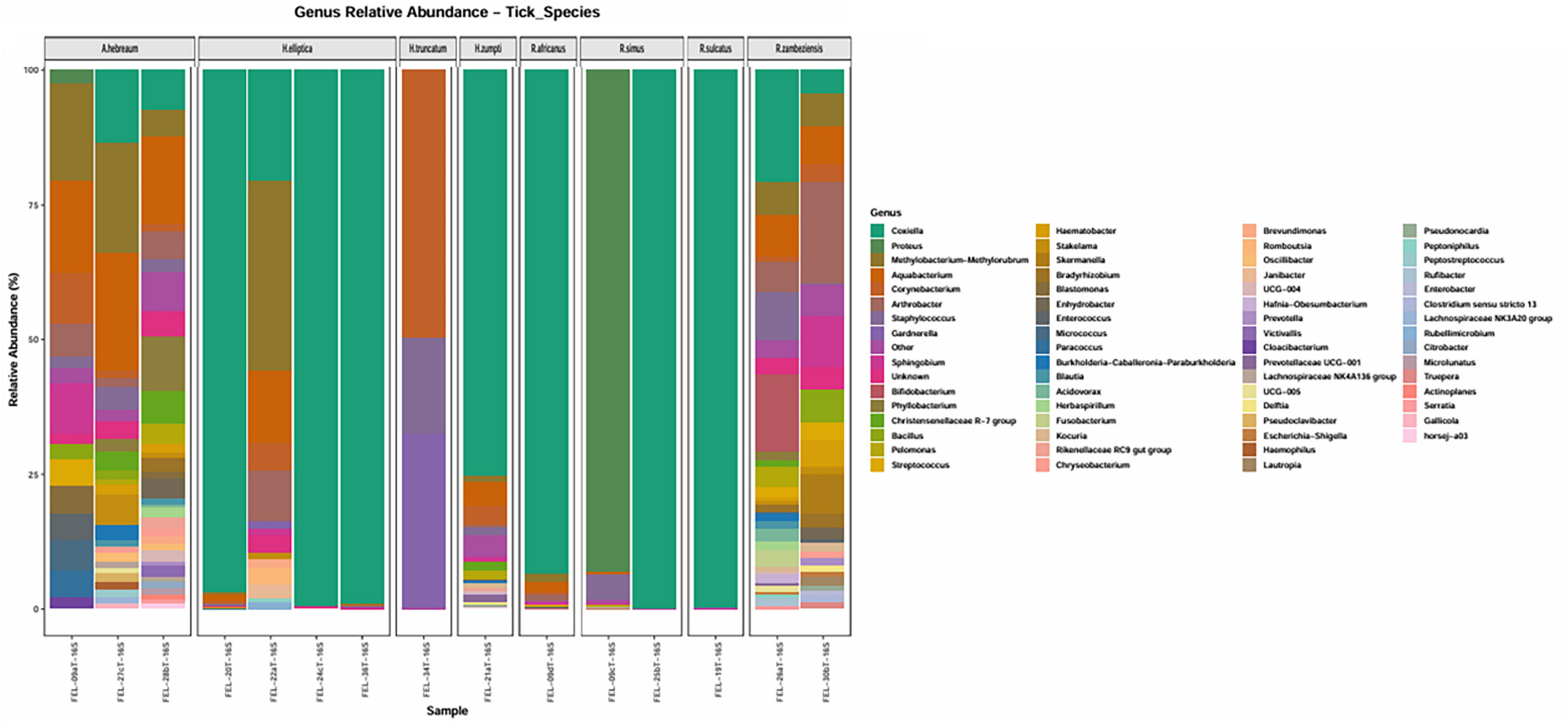
The relative abundance of bacterial genera across different tick species.

With respect to the genus level, there were substantial differences in the composition of the microbial communities among different tick species. Specifically, the most abundant genus among ticks was *Coxiella*, with 79.02%, 75.35%, 93.35%, and 99.88% occurrence in *Ha. elliptica*, *Ha. zumpti*, *R. africanus*, and *R. sulcatus*, respectively. Conversely, *A. hebraeum* had a diverse microbial community dominated by *Aquabacterium* and *Methylobacterium-Methylorubrum* genera, whereas a high abundance of *Corynebacterium*, *Gardnerella*, and *Staphylococcus* was found in *H. truncatum*. Additionally, co-dominant presence of *Coxiella* and *Proteus* genera was found in *R. simus*, however, *R. zambeziensis* represented the highest heterogeneity in microbiomes, Figure 8.

### Alpha-diversity measures between the host and the tick

**FIGURE 9:**
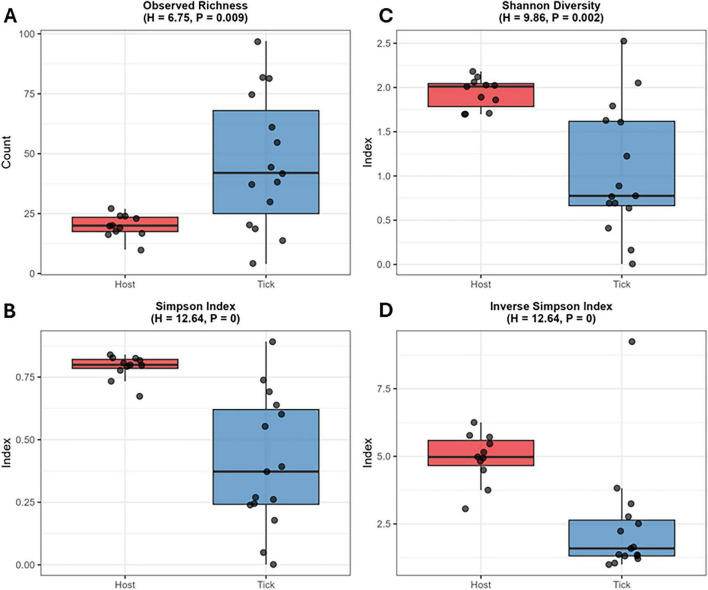
Bacterial richness, diversity, and composition similarity between samples according to sample type. **(A)** Boxplots illustrating the observed diversity index values at the ASV level. **(B)** Simpson diversity index values at the ASV level. **(C)** Shannon diversity index values. **(D)** Inverse Simpson diversity index values at the ASV level.

Alpha diversity and richness estimates between the host and tick varied significantly on all indices evaluated (Figure 9). The Shannon diversity estimate varied significantly between the two groups (Kruskal-Wallis, Kruskal-Wallis *H* = 9.86, *p* = 0.002), with host samples having a higher Shannon diversity compared to tick samples. In addition, Simpson’s diversity measure varied significantly between the two sample groups (Kruskal-Wallis *H* = 12.64, *p* = 0), with host samples having higher Simpson index scores. The inverse Simpson index also revealed a highly significant difference between sample types (Kruskal-Wallis *H* = 12.86, *p* = 0), with hosts demonstrating greater community diversity compared to ticks. In contrast, observed richness differed significantly between hosts and ticks (Kruskal-Wallis *H* = 6.75, *p* = 0,009), with tick samples having higher ASV richness than host samples. Overall, these findings suggest that although tick microbiomes contained a greater number of observed taxa, host microbiomes were characterized by higher diversity and community evenness (Figures 9A–D).

### Alpha-diversity measures across the sexes

**FIGURE 10:**
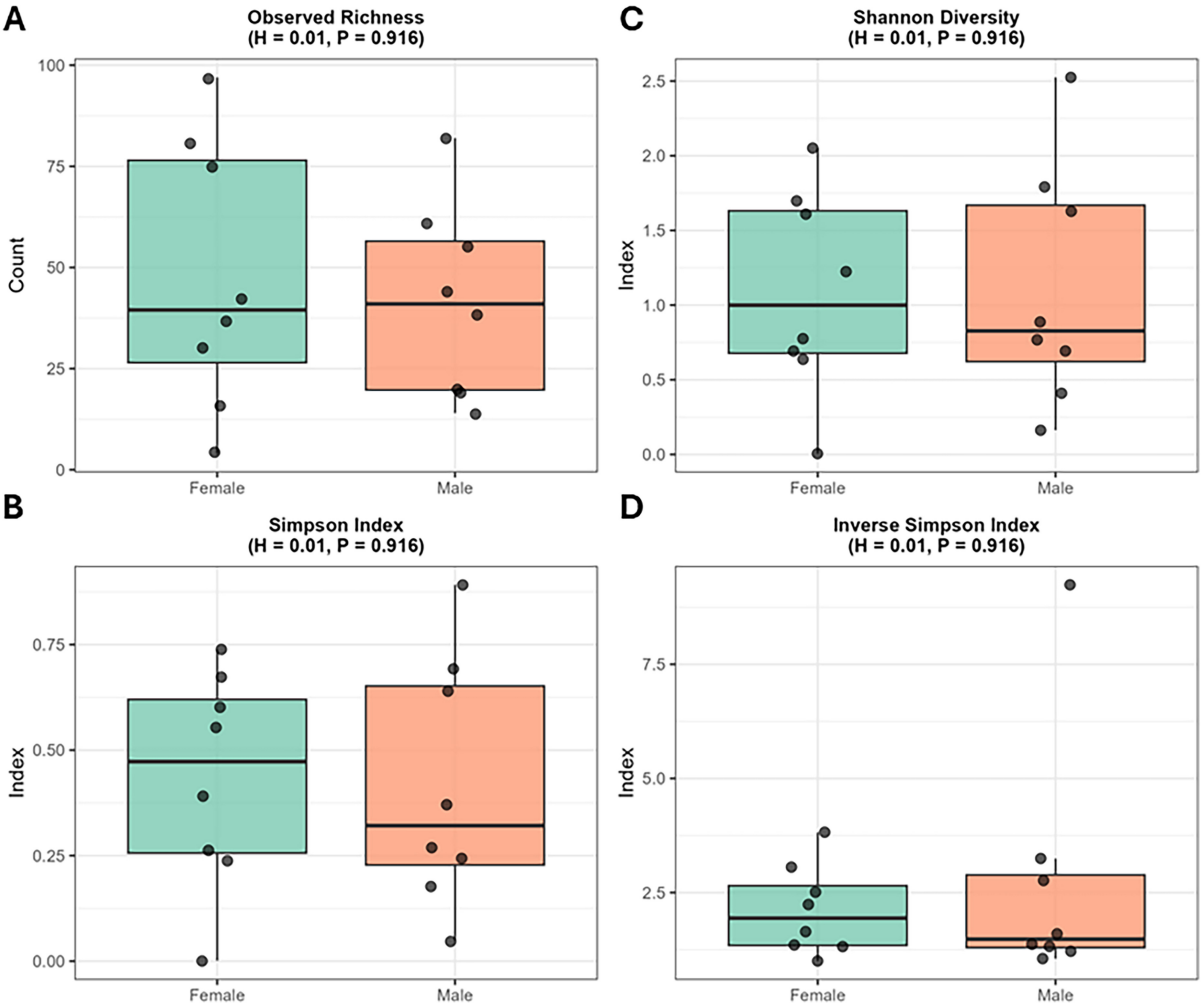
Bacterial richness, diversity, and composition similarity between samples according to sex. **(A)** Boxplots illustrating the observed diversity index values at the ASV level. **(B)** Simpson diversity index values at the ASV level. **(C)** Shannon diversity index values. **(D)** Inverse Simpson diversity index values at the ASV level.

Bacterial alpha-diversity variations between sexes were not statistically significant, according to estimates of indices measuring richness and evenness, including the Shannon, Simpson, Inverse Simpson, and Observed ASVs indices. The Shannon diversity index did not differ significantly between female and male samples (Kruskal-Wallis *H* = 0.01, *p* = 0.916). Similarly, no significant differences were observed for the Simpson diversity index (Kruskal-Wallis *H* = 0.01, *p* = 0.916) or the Inverse Simpson index (Kruskal-Wallis *H* = 0.01, *p* = 0.916). Consistent with these findings, observed richness (Observed ASVs) also showed no significant variation between sexes (Kruskal-Wallis *H* = 0.01, *p* = 0.916). Overall, these results show that there were no statistically significant differences in bacterial alpha-diversity based on indices of bacterial richness and evenness (Figures 10A–D).

### Alpha-diversity measures across the provinces

**FIGURE 11:**
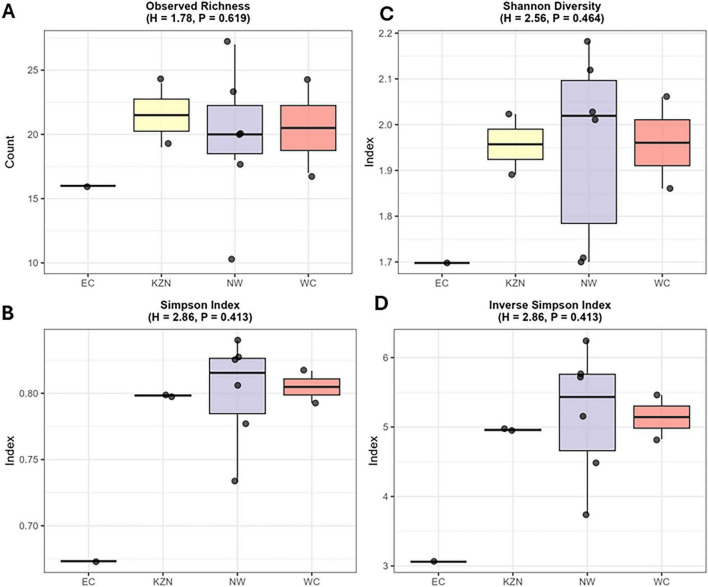
Bacterial richness, diversity, and composition similarity between samples according to province. **(A)** Boxplots illustrating the observed diversity index values at the ASV level. **(B)** Simpson diversity index values at the ASV level. **(C)** Shannon diversity index values. **(D)** Inverse Simpson diversity index values at the ASV level.

The alpha-diversity indices for different measures of richness and evenness such as Shannon, Simpson, Inverse Simpson, and Observed ASVs indices revealed that there were no statistically significant differences in bacterial alpha-diversity between provinces. Specifically, the value of Shannon index was not significant between different provinces (Kruskal-Wallis *H* = 2.56, *p* = 0.464). Moreover, the value of Simpson index between the studied provinces was not significant (Kruskal-Wallis *H* = 2.86, *p* = 0.413), and the same applies to the Inverse Simpson index, where no significant differences between the values were noted (Kruskal-Wallis *H* = 2.86, *p* = 0.413). Furthermore, richness was similarly not significantly different between the studied provinces (Observed ASVs; Kruskal-Wallis *H* = 1.78, *p* = 0.619), Figures 11A–D.

### Alpha-diversity measures across tick species

**FIGURE 12:**
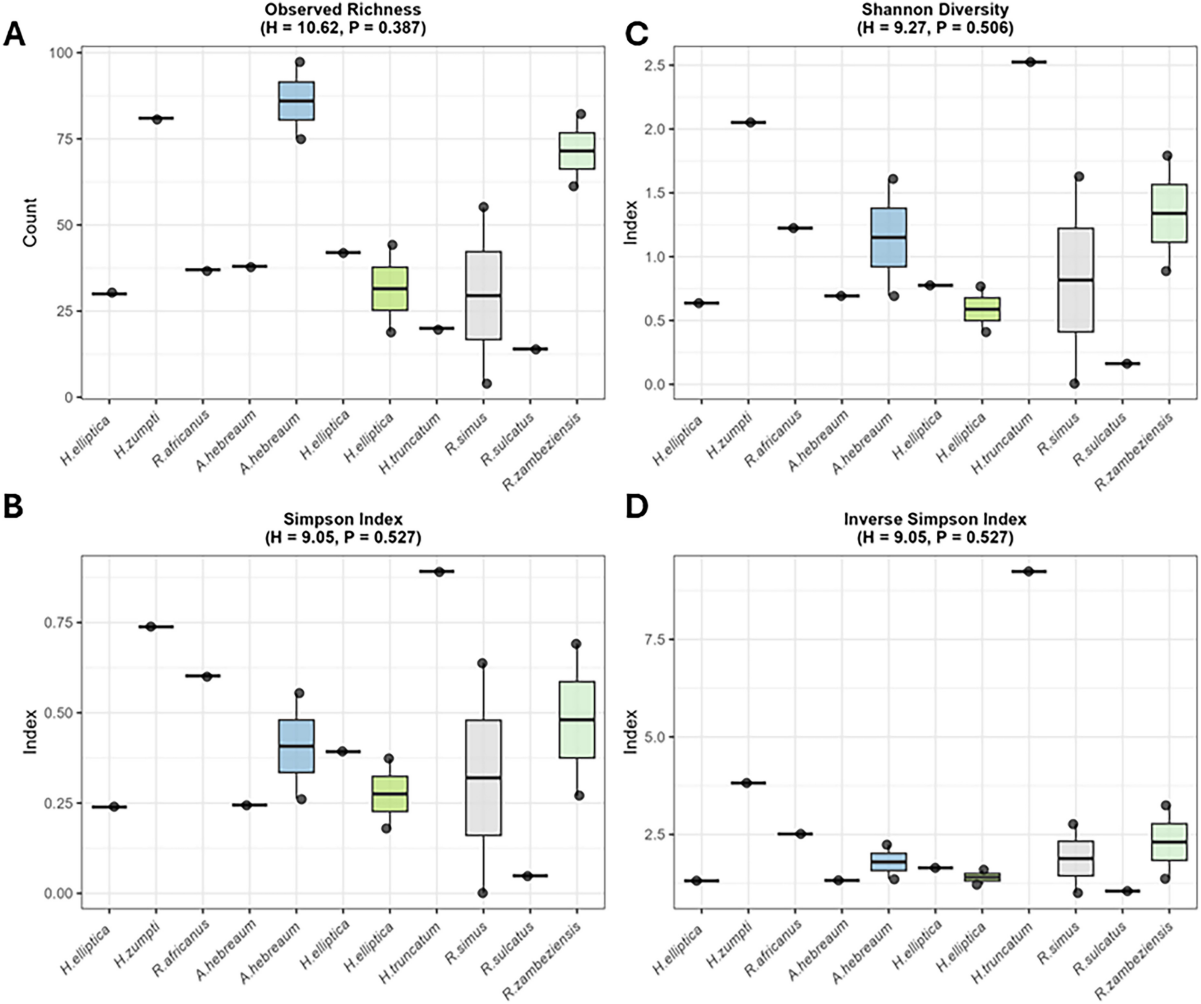
Bacterial richness, diversity, and composition similarity between samples according to tick species. **(A)** Boxplots illustrating the observed diversity index values at the ASV level. **(B)** Simpson diversity index values at the ASV level. **(C)** Shannon diversity index values. **(D)** Inverse Simpson diversity index values at the ASV level.

Estimates of indices measuring richness and evenness, such as Shannon, Simpson, Inverse Simpson, and Observed ASVs, showed that there was no statistical significance in the alpha-diversity of bacteria across different tick species. The Shannon diversity index showed no significant difference among species (Kruskal-Wallis *H* = 9.27, *p* = 0.506). Likewise, the Simpson diversity index showed that there were no statistically significant differences among the species (Kruskal-Wallis *H* = 9.05, *p* = 0.527); consistent results were observed for Inverse Simpson, which also showed no significant differences among the groups (Kruskal-Wallis *H* = 1.78, *p* = 0.619). Observed richness (Observed ASVs) was also consistent with the above findings in that there was no statistical significance in the difference between the tick species (Kruskal-Wallis *H* = 9.05, *p* = 0.527). All the above results point to similar richness and diversity among all tick species (Figures 12A–D).

### Beta-diversity measures in sample-type, sex, tick species, and province

**FIGURE 13:**
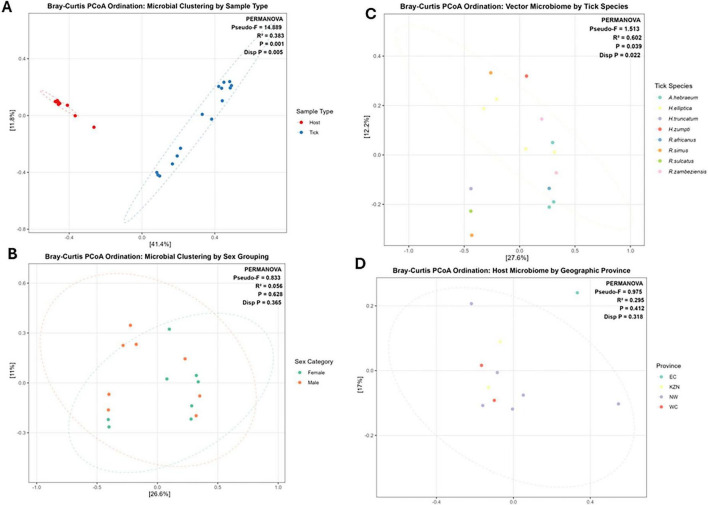
Principal Coordinates Analysis (PCoA) based on weighted UniFrac distances illustrating variation in bacterial composition. **(A)** Clustering patterns according to sample type. **(B)** Variation according to host sex. **(C)** Variation among tick species. **(D)** Variation across provinces. Sample type exerted the strongest influence on clustering, whereas province, host sex, and tick species contributed minimally to the observed variation.

The analysis of PCoA of Bray-Curtis similarity matrices demonstrated that the sample type is the primary factor affecting microbiota structure, with host and tick samples exhibiting significant differences in beta diversity (PERMANOVA, R^2^ = 0.383, *P* = 0.001). In contrast, microbial community composition did not differ significantly among provinces (R^2^ = 0.295, *P* = 0.412) or between male and female ticks (R^2^ = 0.056, *P* = 0.628), with substantial overlap observed among groups. The species explained a significant amount of variance in community structure (R^2^ = 0.602), though the difference was statistically significant (*P* = 0.039), indicating the existence of some differences in the composition of microbial communities depending on tick species. Thus, the main driving force in the structure of the bacterial communities in ticks is the sample source (ticks vs. host), while other variables have no impact or have a minor impact on community composition, Figures 13A–D.

### Bacterial tick-borne species of potential medical and veterinary importance

Several bacterial genera of potential medical and veterinary relevance were detected in both tick and host samples, including *Coxiella*, *Rickettsia*, *Anaplasma*, and *Candidatus Cryptoplasma*, although all except *Coxiella* occurred at relatively low abundances. Multiple ASVs assigned to the genus *Coxiella* were identified in both ticks and hosts, with sequence similarities suggesting the presence of both *Coxiella*-like endosymbionts (CLEs) and bacteria closely related to previously described *Coxiella* species. Most *Coxiella* ASVs detected in ticks showed high similarity to CLEs previously reported from *Rhipicephalus sanguineus*, supporting the widespread occurrence of these endosymbionts in tick microbiomes.

In addition, several ASVs belonging to the genus *Rickettsia* were detected in tick samples and exhibited high sequence similarity to previously described *Rickettsia* spp. Similarly, an ASV assigned to the genus *Anaplasma* was identified in host samples and showed high sequence identity to an uncultured *Anaplasma* species. Furthermore, multiple ASVs belonging to *Candidatus Cryptoplasma* were detected in tick DNA, with the closest matches corresponding to previously reported *Ca. Cryptoplasma* taxa. While these findings demonstrate the presence of genera that include recognized tick-borne bacteria, the use of 16S rRNA gene sequencing does not permit definitive species-level identification or confirmation of pathogenicity. Consequently, the detected taxa should be interpreted as evidence of the presence of these genera rather than confirmation of specific pathogenic species.

## Discussion

Cheetah populations that are kept in captivity are an important part of strategies aimed at their conservation and long-term management. However, enclosures may potentially increase the risk of tick infestations and tick-borne diseases (TBDs), thereby threatening animal health and welfare (Naude et al., 2020; Dickman et al., 2018). This study provides the first characterization of blood- and tick-associated bacterial communities in captive cheetahs in South Africa using full-length 16S rRNA sequencing. This approach can generate insights into factors that influence their health and fitness while directly informing and strengthening conservation practices.

Previous studies have demonstrated that microbiome composition is influenced by interactions between the ticks, their vertebrate hosts and the surrounding environment, yielding distinct microbial communities across different geographical regions (Hawlena et al., 2013; Narasimhan and Fikrig, 2015). Therefore, it is important to understand these spatial patterns to advance knowledge on the ecology of ticks and ultimately develop better ways of controlling TBPs. Unfortunately, less is currently known about the tick microbiome and there are currently no reports on blood-associated microbial communities in either captive or free-ranging cheetahs in South Africa.

Our results revealed that cheetahs in captivity harbor diverse bacterial communities, and significant variation in microbial diversity (beta diversity) between host-associated (blood) and tick-associated microbial communities (*p* < 0.05), indicating that these microbial assemblages within these hosts and ticks are compositionally distinct rather than randomly structured. This pattern suggests that both the tick and the host environments act as strong ecological filters, shaping microbial community assembly through differences in physiology, immune pressure, diet, and habitat (Narasimhan et al., 2021). In contrast to our study, beta diversity analysis and ordination revealed little overlap between the sample types collected from blood, ticks and tissue samples of wildlife (Egan et al., 2021). Several other studies also found that there is little correlation between host skin or blood and the microbiomes of hard ticks (Hawlena, et al., 2013; Rynkiewicz, et al., 2015; Zolnik et al., 2016).

We observed little to no variation of the beta diversity affected by geographic region and sex. This is consistent with the studies by Kueneman et al. (2021) and Al Masri et al. (2025) who also found that sex does not play a major role in structuring microbial profiles between male and female ticks from camels.

Our study also reported a significantly different alpha diversity between the two sample types (ticks and the host blood), consistent with a study by Al Masri et al. (2025). Although not significant, the male ticks in our study exhibited higher microbial diversity composition and richness across all sampled locations. Our findings contradict previously reported studies on a diverse bacterial taxon with a comparable or higher diversity than some vertebrate host-associated microbiomes (Carpi et al., 2011; Krawczyk et al., 2022). Understanding these differences is crucial for developing targeted strategies for TBDs and highlights the importance of considering sex as a biological variable in vector microbiome studies (Al Masri et al., 2025). Geographical locations did not drive tick richness as much as the sample type intrinsic factor. These findings are consistent with previous work that indicated that alpha diversity does not differ across various locations (Zolnik et al., 2016). Although the translocation histories of the animals were not available, the observed results could also be due to the constant movement of the animals for conservation purposes.

Proteobacteria was the dominant phylum in both hosts and ticks, while Actinobacteriota and Firmicutes were also prominent components of the microbiome. The predominance of Proteobacteria is consistent with reports from other tick microbiome studies and likely reflects the importance of this phylum in host-microbe interactions and tick-associated symbioses (Narasimhan et al., 2014; Lalzar et al., 2014). Notably, Proteobacteria often harbor endosymbionts or interact with tick-borne pathogens like *Rickettsia*, which was observed in this study.

The presence and distribution of certain genera of bacteria in this study was consistent with the results observed in other studies (Narasimhan and Fikrig, 2015; Bonnet et al., 2017; Greay et al., 2018; Egan et al., 2021). The microbiome community associated with the host was heavily dominated by *Sphingobium*, followed by *Bacillus*, *Stenotrophomonas*, *Leifsonia*, and *Staphylococcus*. This microbial community structure appears highly uncharacteristic of obligate carnivores, implying that the microbiota associated with the host may be a combination of environmental, dietary, and host-associated bacteria. Similarly, the presence of *Bradyrhizobium*, *Methylobacterium-Methylorubrum*, and *Cupriavidus* also hints at environmental origin, since these genera are well-known soil-plant bacteria. Such taxa have been reported in wildlife microbiomes exposed to natural environments, particularly where diet and water sources contribute transient microbial input (Maurice et al., 2015).

The identification of *Bifidobacterium* and *Atopobium* suggests that there might be an indication of small input from gut-associated fermenters, rarely encountered in carnivorous mammals. In addition, the presence of *Bartonella* and *Anaplasma* in the host may indicate that they are vector-associated bacteria or blood-associated ones, which are found in wild carnivorous mammals because of the attack by ticks or blood-sucking arthropods (Chomel and Kasten, 2010; Kelly et al., 2014). Conversely, *Pelomonas*, *Brevundimonas*, and *Novosphingobium* detected in low abundance (<1%) can indicate that the identified microbial profile corresponds to water or environmental microorganisms. These could suggest that the indicated microbial profile is characterized by considerable environmental and probably food-based microbial input.

The tick microbiome was mainly comprised of bacteria in the genus *Coxiella*. The widespread occurrence and high relative abundance of *Coxiella* across tick species is consistent with the recognized role of *Coxiella*-like endosymbionts in tick nutrition, development, and reproduction (Klyachko et al., 2007; Zhong et al., 2007; Bonnet et al., 2017). However, species-level identification and functional inference remain limited when based solely on 16S rRNA metabarcoding.

Identification of *Proteus*, *Staphylococcus*, *Corynebacterium*, and *Enterococcus* bacteria may imply that the organisms were involved in the process of feeding by ticks through contact with mammals. These are usually common microbes present in most ticks and are regarded as either transient or opportunistically acquired once the ticks feed on the host animals (Narasimhan et al., 2014; Narasimhan and Fikrig, 2015). The presence of bacteria in the environment, such as *Methylobacterium-Methylorubrum*, *Aquabacterium*, *Bradyrhizobium*, is possibly caused by tick acquisition of the bacteria from their vegetation areas (Abraham et al., 2017).

In addition to the environmental bacteria, several genera containing known human and animal pathogens were detected at low abundance in host samples, including *Bartonella*, *Anaplasma*, *Escherichia-Shigella*, *Enterobacter*, *Streptococcus*, *Corynebacterium*, *Finegoldia*, and *Staphylococcus*. While these genera include species associated with zoonotic, and opportunistic infections, their detection does not indicate active infection or disease (Murdoch, 1998; Chomel and Kasten, 2010; Kocan et al., 2010; Tenaillon et al., 2010; Tong et al., 2015).

The tick microbiome also comprised low-abundance genera that include known pathogens, such as *Escherichia-Shigella*, *Enterobacter*, *Streptococcus*, *Staphylococcus*, *Corynebacterium* and *Haemophilus*. However, the community was dominated by *Coxiella*, a common tick endosymbiont involved in nutritional supplementation (Zhong et al., 2007; Bonnet et al., 2017). Overall, the detection of potential pathogen-associated genera should be interpreted cautiously, their presence may reflect transient, environmental or non-pathogenic bacterial signatures rather than active infection (Narasimhan and Fikrig, 2015).

Across all provinces, host microbiomes were dominated by environmental genera such as *Sphingobium*, *Bacillus*, and *Leifsonia*, indicating substantial environmental influence (soil, vegetation) on microbial community composition (Narasimhan and Fikrig, 2015; Bonnet et al., 2017). Although some provincial variation was observed, including the detection of *Anaplasma* and *Bartonella* in certain regions, these findings likely reflect ongoing microbial acquisition from environmental and host-associated sources during tick development and feeding. The identification of reflecting coexistence of environmental taxa with bacteria linked to tick-vertebrate transmission cycles, although its presence indicates potential circulation rather than confirmed infection (Kocan et al., 2004, Abraham et al., 2017). The bacterial community composition varied among tick species, indicating species-specific microbiome structuring. However, *Coxiella* was the dominant genus in most species, accounting for more than 75% of sequences in *Ha. elliptica*, *Ha. zumpti*, *R. africanus*, and *R. sulcatus*. This widespread dominance supports its established role as a core tick endosymbiont involved in nutrition, development, and reproduction (Machado-Ferreira et al., 2016; Bonnet et al., 2017). In contrast, *A. hebraeum*, *Hy. truncatum*, *R. simus*, and *R. zambeziensis* exhibited more diverse bacterial communities, characterized by genera such as *Aquabacterium*, *Methylobacterium-Methylorubrum*, *Corynebacterium*, *Gardnerella*, *Staphylococcus*, and *Proteus*. These taxa are commonly associated with environmental reservoirs, vertebrate hosts, or host skin microbiota, suggesting that habitat exposure, host interactions, and feeding ecology contribute substantially to microbiome composition (Narasimhan and Fikrig, 2015; Abraham et al., 2017).

*Coxiella* was the predominant genus in both sexes, accounting for 50.9% and 40.5% of the bacterial community in females and males, respectively. The prevalence of *Coxiella* is consistent with its established role as a widespread tick endosymbiont that contributes essential nutrients required for tick development and reproduction (Machado-Ferreira et al., 2016; Bonnet et al., 2017). The higher abundance of *Coxiella* in females may reflect the greater nutritional demands associated with reproduction, as maternally inherited endosymbionts are often more abundant in female ticks and play a critical role in reproductive fitness (Zhong et al., 2007).

Apart from *Coxiella*, sex-specific differences in bacterial composition were observed, with females haboring higher abundance of environmental bacteria such as *Methylobacterium-Methylorubrum*, *Aquabacterium*, *Leifsonia*, and *Sphingobium*, while males had higher abundance of *Proteus*, *Corynebacterium*, *Staphylococcus*, and *Gardnerella*. These differences may reflect variation in host interactions, physiology, or environmental exposure (Narasimhan and Fikrig, 2015; Abraham et al., 2017). Nevertheless, a *Coxiella*-dominated core microbiome was conserved across sexes and species, consistent with previous reports in *Amblyomma* and *Haemaphysalis* ticks, where *Coxiella*-like endosymbionts are a major component of the microbiome (Budachetri et al., 2014; Kumar et al., 2022; Kim et al., 2021; Jiang et al., 2024; Ponnusamy et al., 2024). Other genera like *Staphylococcus*, and *Pseudomonas* represented only 10% of bacteria in total (Ponnusamy et al., 2024) and are considered environmentally acquired organisms. Collectively, these findings suggest that the tick microbiome is predominantly composed of symbiont-associated bacteria, although further species-specific studies are needed to confirm these patterns.

The predominance of *Coxiella*-like endosymbionts observed in this study is consistent with previous reports in *Hyalomma* and *Rhipicephalus* ticks, where these bacteria constitute a major component of the tick microbiome (Guizzo et al., 2022; Al Masri et al., 2025). In high throughput microbiome studies, it was confirmed that *Coxiella*-like endosymbionts might dominate *Rh. Microplus* bacterial community, contributing major proportion of 16S sequencing in early developmental stages (Guizzo et al., 2022). *Coxiella*-like endosymbionts are vertically transmitted and are involved in crucial physiological processes in ticks (Guizzo et al., 2022). A literature survey on *Hyalomma* ticks’ microbiome composition indicated that environmental and opportunistic bacterial genus, such as is *Pseudomonas* is common alongside other taxa, namely *Bacillus*, *Flavobacterium*, *Staphylococcus*, and *Corynebacterium* (Al Masri et al., 2025), consistent with our results.

A major limitation of this study was the relatively small and uneven sample size, resulting from opportunistic sampling, DNA quality constraints, sequencing costs, and the exclusion of low-quality or low-depth samples. These factors reduced the statistical power for comparisons across provinces, tick species, sexes, host categories, and life stages; therefore, subgroup analyses should be interpreted with caution. Although contamination controls and decontamination procedures were implemented, low-biomass microbiome studies remain susceptible to background contamination. Consequently, the findings should be regarded as exploratory and hypothesis-generating, highlighting the need for larger, systematically collected datasets to validate the observed patterns.

## Conclusion

This study provides the first comprehensive microbiome-based characterization of blood- and tick-associated bacterial communities in captive cheetahs from selected conservation facilities in South Africa. The application of advanced metabarcoding, through PacBio sequencing approach, identified numerous previously uncharacterized species across all the evaluated variables, including established tick-associated endosymbionts and reported zoonotic bacterial pathogens, although the infection status and pathogenic relevance of these bacteria in the sampled hosts remain unknown and several previously uncharacterized or poorly described taxa, highlighting the immense microbial diversity beyond the well-characterized taxa in curated databases. Additionally, the SILVA database is constrained by high sequence conservation within this genus, which can lead to misclassification. The observed microbial distribution provides valuable insight into the potential ecological roles of dominant phyla of veterinary and public health importance within ticks from captive cheetahs and offers a foundation for future investigations into tick-microbe-pathogen interactions in threatened wildlife. Future studies should be directed toward enhancing genome reconstruction by combining long- and short-read assemblies, as well as developing metagenome-assembled genomes that would improve the taxonomic and functional characterization of unknown microbial species as the 16S rRNA metabarcoding approach does not reliably provide species-level resolution. Expanding databases through functional studies would improve understanding of microbial roles in natural ecosystems. Given the limited sample size and exploratory nature of the study, these findings provide a baseline for future investigations of host-tick microbial interactions in cheetahs and other threatened wildlife. Future studies incorporating larger sample sizes and complementary genomic approaches, such as targeted PCR assays, metagenomics, or whole-genome sequencing, will be required to better characterize microbial diversity and assess the ecological and health significance of.

## Data Availability

The datasets presented in this study can be found in online repositories. The names of the repository/repositories and accession number(s) can be found below: https://www.ncbi.nlm.nih.gov/, (SAMN54241566 – SAMN54241596), PRJNA1392041.

## Supplementary material

Supplementary Figure 1 Sequencing depth across all samples showing the total number of reads retained per sample after quality filtering. Negative controls are highlighted in red, while biological samples are shown in turquoise.

Supplementary Figure 2 **(A)** Prevalence-based decontamination analysis identified contaminant and **(B)** retained ASVs based on statistical probability scores. **(C)** Contaminant ASVs were removed prior to downstream analyses, with the red dashed line indicating the selected classification threshold (*p* = 0.5).

Supplementary Figure 3 Comparison of total read counts per sample before (blue) and after (gray) filtering for contaminants using decontam.

Supplementary Figure 4 **(A)** Rarefaction curves showing ASV(s) richness as a function of sequencing depth before rarefaction **(A)** and after rarefaction **(B)**.

## References

[B1] Abraham N. M. Liu L. Jutras B. L. Yadav A. K. Narasimhan S. Gopalakrishnan V. et al. (2017). Pathogen-mediated manipulation of arthropod microbiota to promote infection. *Proc. Natl. Acad. Sci. U. S. A*. 114 E781–E790. 10.1073/pnas.1613422114 28096373 PMC5293115

[B2] Al Masri M. T. Ali A. S. Vijayan R. Muzaffar S. B. Al-Deeb M. A. (2025). Microbiome variation between male and female *Hyalomma dromedarii* ticks from camels in the UAE. *Sci. Rep*. 15:30990. 10.1038/s41598-025-16581-6 40847065 PMC12373762

[B3] Amato K. R. Yeoman C. J. Kent A. Righini N. Carbonero F. Estrada A. et al. (2013). Habitat degradation impacts black howler monkey (*Alouatta pigra*) gastrointestinal microbiomes. *ISME J*. 7 1344–1353. 10.1038/ismej.2013.16 23486247 PMC3695285

[B4] Banks N. (1908). *A Revision of the Ixodoidea, or Ticks, of the United States.* Washington, DC: U.S. Department of Agriculture, Bureau of Entomology.

[B5] Black W. C. Piesman J. (1994). Phylogeny of hard- and soft-tick taxa (Acari: Ixodida) based on mitochondrial 16S rDNA sequences. *Proc. Natl. Acad. Sci. U. S. A*. 91 10034–10038. 10.1073/pnas.91.21.10034 7937832 PMC44952

[B6] Bonnet S. I. Binetruy F. Hernández-Jarguín A. M. Duron O. (2017). The tick microbiome: Why non-pathogenic microorganisms matter in tick biology and pathogen transmission. *Front. Cell Infect. Microbiol*. 7:236. 10.3389/fcimb.2017.00236 28642842 PMC5462901

[B7] Budachetri K. Browning R. E. Adamson S. W. Dowd S. E. Chao C. C. Ching W. M. et al. (2014). An insight into the microbiome of the *Amblyomma maculatum* (Acari: Ixodidae). *J. Med. Entomol*. 51 119–129. 10.1603/me12223 24605461 PMC3956751

[B8] Callahan B. J. McMurdie P. J. Rosen M. J. Han A. W. Johnson A. J. Holmes S. P. (2016). DADA2: high-resolution sample inference from Illumina amplicon data. *Nat. Methods* 13 581–583. 10.1038/nmeth.3869 27214047 PMC4927377

[B9] Carpi G. Cagnacci F. Wittekindt N. E. Zhao F. Qi J. Tomsho L. P. et al. (2011). Metagenomic profile of the bacterial communities associated with *Ixodes ricinus* ticks. *PLoS One* 6:e26014. 10.1371/journal.pone.0025604 22022422 PMC3192763

[B10] Castillo D. J. Rifkin R. F. Cowan D. A. Potgieter M. (2019). The healthy human blood microbiome: fact or fiction? *Front. Cell Infect. Microbiol*. 9:148. 10.3389/fcimb.2019.00148 31139578 PMC6519389

[B11] Chomel B. B. Kasten R. W. (2010). Bartonellosis, an increasingly recognized zoonosis. *J. Appl. Microbiol*. 109 743–750. 10.1111/j.1365-2672.2010.04679.x 20148999

[B12] Davis N. M. Proctor D. M. Holmes S. P. Relman D. A. Callahan B. J. (2018). Simple statistical identification and removal of contaminant sequences in marker-gene and metagenomics data. *Microbiome* 6:226. 10.1186/s40168-018-0605-2 30558668 PMC6298009

[B13] de Cock M. Fonville M. de Vries A. Bossers A. van den Bogert B. Hakze-van der Honing R. et al. (2022). Screen the unforeseen: microbiome-profiling for detection of zoonotic pathogens in wild rats. *Transbound. Emerg. Dis*. 69 3881–3895. 10.1111/tbed.14759 36404584 PMC10099244

[B14] Dickman A. Rust N. A. Boast L. K. Wykstra M. Richmond-Coggan L. Klein R. et al. (2018). “The costs and causes of human-cheetah conflict on livestock and game farms,” in *Cheetah Range Wide Status and Distribution*, ed. Nyhus P. J. (New York, NY: Academic Press).

[B15] Durant S. M. Groom R. Ipavec A. Mitchell N. Khalatbari L. (2024). *Acinonyx jubatus* (amended version of 2023 assessment). *IUCN Red List Threat.Species* 2024:e.T219A259025524.

[B16] Durant S. M. Mitchell N. Groom R. Pettorelli N. Ipavec A. Jacobson A. P. et al. (2017). The global decline of cheetah Acinonyx jubatus and what it means for conservation. *Proc. Natl. Acad. Sci. U. S. A*. 114 528–533. 10.1073/pnas.1611122114 28028225 PMC5255576

[B17] Egan S. L. Taylor C. L. Banks P. B. Northover A. S. Ahlstrom L. A. Ryan U. M. et al. (2021). The bacterial biome of ticks and their wildlife hosts at the urban-wildland interface. *Microb Genom*. 7:000730. 10.1099/mgen.0.000730 34913864 PMC8767321

[B18] Folmer O. Black M. Hoeh W. Lutz R. Vrijenhoek R. (1994). DNA primers for amplification of mitochondrial cytochrome c oxidase subunit I from diverse metazoan invertebrates. *Mol. Mar. Biol. Biotechnol.* 3 294–299. 7881515

[B19] Greay T. L. Gofton A. W. Paparini A. Ryan U. M. Oskam C. L. Irwin P. J. (2018). Recent insights into the tick microbiome gained through next-generation sequencing. *Parasit Vect*. 11:12. 10.1186/s13071-017-2550-5 29301588 PMC5755153

[B20] Guizzo M. G. Tirloni L. Gonzalez S. A. Farber M. D. Braz G. Parizi L. F. et al. (2022). Coxiella endosymbiont of Rhipicephalus microplus modulates tick physiology with a major impact in blood feeding capacity. *Front. Microbiol*. 13:868575. 10.3389/fmicb.2022.868575 35591999 PMC9111531

[B21] Hawlena H. Rynkiewicz E. Toh E. Alfred A. Durden L. A. Hastriter M. W. et al. (2013). The arthropod, but not the vertebrate host or its environment, dictates bacterial community composition of fleas and ticks. *ISME J*. 7 221–223. 10.1038/ismej.2012.71 22739493 PMC3526175

[B22] Honneffer J. B. Minamoto Y. Suchodolski J. S. (2014). Microbiota alterations in acute and chronic gastrointestinal inflammation of cats and dogs. *World J. Gastroenterol*. 20 16489–16497. 10.3748/wjg.v20.i44.16489 25469017 PMC4248192

[B23] Horak I. G. Heyne H. Williams R. Gallivan G. J. Spickett A. M. Bezuidenhout J. D. et al. (2018). The ixodid ticks (Acari: Ixodidae) of southern Africa. *Onderstepoort J. Vet. Res.* 85 1–16. 10.1007/978-3-319-70642-9

[B24] Janeczko S. Atwater D. Bogel E. Greiter-Wilke A. Gerold A. Baumgart M. et al. (2008). The relationship of mucosal bacteria to duodenal histopathology, cytokine mRNA, and clinical disease activity in cats with inflammatory bowel disease. *Vet. Microbiol*. 128 178–193. 10.1016/j.vetmic.2007.10.014 18054447

[B25] Jeo R. M. Schmidt-Küntzel A. Ballou J. D. Sanjayan M. S. (2018). “Drivers of habitat loss and fragmentation: implications for the design of landscape linkages for cheetahs,” in *Cheetahs: Biology and Conservation*, ed. Nyhus P. (New York, NY: Academic Press), 137–149. 10.1016/B978-0-12-804088-1.00010-1

[B26] Jiang S. Kang M. Li Z. Han X. Chen C. He S. et al. (2024). The impact of bloodmeal and geographic region on the richness, diversity, and function of internal microbial community in *Haemaphysalis qinghaiensis* from the Qinghai province, China. *Heliyon* 10:e35429. 10.1016/j.heliyon.2024.e35429 39165970 PMC11334854

[B27] Kelly P. Marabini L. Dutlow K. Zhang J. Loftis A. Wang C. (2014). Molecular detection of tick-borne pathogens in captive wild felids, Zimbabwe. *Parasit Vect*. 7:514. 10.1186/s13071-014-0514-6 25404084 PMC4243927

[B28] Kendon T. A. Pereira C. L. Pereira H. Brown K. Gaynor D. Briers-Louw W. D. (2025). Incidents of high tick load in injured cheetahs after reintroduction into a tropical ecosystem. *Onderstepoort J. Vet. Res*. 92 e1–e5. 10.4102/ojvr.v92i1.2206 40336442 PMC12067641

[B29] Khoza B. L. Byaruhanga C. Makgabo S. M. Nyangiwe N. Mnisi T. Nxumalo S. et al. (2024). Tick distribution and comparative analysis of bovine blood microbiome in two provinces of South Africa using 16S rRNA PacBio sequencing approach. *Front. Trop. Dis.* 5:1399364. 10.3389/fitd.2024.1399364

[B30] Kim M. Kim J. Y. Yi M. H. Lee I. Y. Yong D. Jeon B. Y. et al. (2021). Microbiome of *Haemaphysalis longicornis* Tick in Korea. *Korean J. Parasitol*. 59 489–496. 10.3347/kjp.2021.59.5.489 34724768 PMC8561044

[B31] Klyachko O. Stein B. D. Grindle N. Clay K. Fuqua C. (2007). Localization and visualization of a coxiella-type symbiont within the lone star tick, *Amblyomma americanum*. *Appl. Environ. Microbiol*. 73 6584–6594. 10.1128/AEM.00537-07 17720830 PMC2075054

[B32] Kocan K. M. de la Fuente J. Blouin E. F. Coetzee J. F. Ewing S. A. (2010). The natural history of Anaplasma marginale. *Vet. Parasitol.* 167, 95–107. 10.1016/j.vetpar.2009.09.012 19811876

[B33] Kocan K. M. de la Fuente J. Blouin E. F. Garcia-Garcia J. C. (2004). *Anaplasma marginale* (Rickettsiales: Anaplasmataceae): recent advances in defining host-pathogen adaptations of a tick-borne rickettsia. *Parasitology* 129(Suppl.), S285–S300. 10.1017/s0031182003004700 15938516

[B34] Kolo A. O. Brayton K. A. Collins N. E. Bastos A. D. S. Matthee S. Gall C. A. et al. (2025). Bacterial blood microbiome of Mastomys rodents: implications for disease spill-over at the animal-human interface within the Bushbuckridge-East community, South Africa. *Front. Cell Infect. Microbiol*. 15:1520086. 10.3389/fcimb.2025.1520086 39963409 PMC11830667

[B35] Krawczyk A. I. Röttjers L. Fonville M. Takumi K. Takken W. Faust K. et al. (2022). Quantitative microbial population study reveals geographical differences in bacterial symbionts of *Ixodes ricinus*. *Microbiome* 10:120. 10.1186/s40168-022-01276-1 35927748 PMC9351266

[B36] Kueneman J. G. Esser H. J. Weiss S. J. Jansen P. A. Foley J. E. (2021). Tick microbiomes in neotropical forest fragments are best explained by tick-associated and environmental factors rather than host blood source. *Appl. Environ. Microbiol*. 87:e02668-20. 10.1128/AEM.02668-20 33514519 PMC8091620

[B37] Kumar D. Sharma S. R. Adegoke A. Kennedy A. Tuten H. C. Li A. Y. et al. (2022). Recently evolved francisella-like endosymbiont outcompetes an ancient and evolutionarily associated coxiella-like endosymbiont in the lone star tick (*Amblyomma americanum*) linked to the alpha-gal syndrome. *Front. Cell Infect. Microbiol.* 12:787209. 10.3389/fcimb.2022.787209 35493735 PMC9039623

[B38] Lalzar I. Friedmann Y. Gottlieb Y. (2014). Tissue tropism and vertical transmission of *Coxiella* in *Rhipicephalus sanguineus* and *Rhipicephalus turanicus* ticks. *Environ. Microbiol.* 16, 3657–3668. 10.1111/1462-2920.12455 24650112

[B39] Lane D. J. (1991). “16S/23S rRNA sequencing,” in *Nucleic Acid Techniques in Bacterial Systematics*, eds Stackebrandt E. Goodfellow M. (New York, NY: John Wiley & Sons), 115–175.

[B40] Ledwaba M. B. Nozipho K. Tembe D. Onyiche T. E. Chaisi M. E. (2022). Distribution and prevalence of ticks and tick-borne pathogens of wild animals in South Africa: a systematic review. *Curr. Res. Parasitol. Vector Borne Dis*. 2:100088. 10.1016/j.crpvbd.2022.100088 35601607 PMC9114622

[B41] Machado-Ferreira E. Vizzoni V. F. Balsemão-Pires E. Moerbeck L. Gazeta G. S. Piesman J. et al. (2016). Coxiella symbionts are widespread into hard ticks. *Parasitol. Res*. 115 4691–4699. 10.1007/s00436-016-5230-z 27595990

[B42] Magliolo M. Naude V. N. van der Merwe V. C. Prost S. Orozco-terWengel P. Burger P. A. et al. (2023). Simulated genetic efficacy of metapopulation management and conservation value of captive reintroductions in a rapidly declining felid. *Anim. Conserv.* 26:263. 10.1111/acv.12821

[B43] Maly M. A. Crosier A. E. Keady M. M. Roberts B. R. Breen M. Muletz Wolz C. R. (2024). Stability of fecal microbiota during degradation in captive cheetahs in the US. *Microbiol. Host* 2:22. 10.1530/MAH-23-0022

[B44] Marnewick K. Beckhelling A. Cilliers D. Lane E. Mills G. Herring K. et al. (2007). The status of the cheetah in South Africa. *CAT News* 3 22–31.

[B45] Maurice C. Knowles S. C. L. Ladau J. Pollard K. S. Fenton A. Pedersen A. B. et al. (2015). Marked seasonal variation in the wild mouse gut microbiota. *ISME J.* 9 2423–2434. 10.1038/ismej.2015.53 26023870 PMC4611506

[B46] McMurdie P. J. Holmes S. (2013). phyloseq: an R package for reproducible interactive analysis and graphics of microbiome census data. *PLoS One* 8:e61217. 10.1371/journal.pone.0061217 23630581 PMC3632530

[B47] Menke S. Meier M. Sommer S. Bunce M. (2015). Shifts in the gut microbiome observed in wildlife faecal samples exposed to natural weather conditions: lessons from time-series analyses using next-generation sequencing. *Methods Ecol. Evol.* 6:1087. 10.1111/2041-210X.12394

[B48] Murdoch D. A. (1998). Gram-positive anaerobic cocci. *Clin. Microbiol. Rev*. 11 81–120. 10.1128/CMR.11.1.81 9457430 PMC121377

[B49] Narasimhan S. Fikrig E. (2015). Tick microbiome: the force within. *Trends Parasitol*. 31 315–323. 10.1016/j.pt.2015.03.010 25936226 PMC4492851

[B50] Narasimhan S. Rajeevan N. Liu L. Zhao Y. O. Heisig J. Pan J. et al. (2014). Gut microbiota of the tick vector *Ixodes scapularis* modulate colonization of the Lyme disease spirochete. *Cell Host Microbe* 15 58–71. 10.1016/j.chom.2013.12.001 24439898 PMC3905459

[B51] Narasimhan S. Swei A. Abouneameh S. Pal U. Pedra J. H. F. Fikrig E. (2021). Grappling with the tick microbiome. *Trends Parasitol*. 37 722–733. 10.1016/j.pt.2021.04.004 33962878 PMC8282638

[B52] Naude V. N. Balme G. A. Rogan M. S. Needham M. D. Whittington-Jones G. Dickerson T. et al. (2020). Longitudinal assessment of illegal leopard skin uses in ceremonial regalia and acceptance of faux alternatives among followers of the Shembe Church, South Africa. *Conserv. Sci. Pract.* 2:e116. 10.1111/csp2.289

[B53] Nyamota R. Middlebrook E. A. Abkallo H. M. Akoko J. Gakuya F. Wambua L. et al. (2025). The Bacterial and pathogenic landscape of African buffalo (*Syncerus caffer*) whole blood and serum from Kenya. *Anim. Microbiome* 7:6. 10.1186/s42523-024-00374-9 39800778 PMC11725222

[B54] Ontiveros-Chacón J. C. García-De La Peña C. Domínguez-Viveros J. Aguilar-Palma G. N. Ávila-Rodríguez V. Estrada-Arellano J. R. et al. (2025). Blood bacterial microbiota of the American bison (*Bison bison*) in Northern Mexico: a reference for health and conservation. *Ruminants* 5:10. 10.3390/ruminants5010010

[B55] Pannoni S. B. Proffitt K. M. Holben W. E. (2022). Non-invasive monitoring of multiple wildlife health factors by fecal microbiome analysis. *Ecol. Evol*. 12:e8564. 10.1002/ece3.8564 35154651 PMC8826075

[B56] Ponnusamy L. Travanty N. V. Watson D. W. Seagle S. W. Boyce R. M. Reiskind M. H. (2024). Microbiome of invasive tick species *Haemaphysalis longicornis* in North Carolina, USA. *Insects* 15:153. 10.3390/insects15030153 38535349 PMC10970973

[B57] Potgieter K. R. O’Riain J. M. Davies-Mostert H. T. Forssman K. (2015). Behavioural cues can be used to predict the outcome of artificial pack formation in African wild dogs (*Lycaon pictus*). *S. Afr. J. Wildl. Res.* 45 215–222. 10.3957/056.045.0215

[B58] Quast C. Pruesse E. Yilmaz P. Gerken J. Schweer T. Yarza P. et al. (2013). The SILVA ribosomal RNA gene database project: improved data processing and web-based tools. *Nucleic Acids Res*. 41 D590–D596. 10.1093/nar/gks1219 23193283 PMC3531112

[B59] Rynkiewicz E. C. Hemmerich C. Rusch D. B. Fuqua C. Clay K. (2015). Concordance of bacterial communities of two tick species and blood of their shared rodent host. *Mol. Ecol*. 24 2566–2579. 10.1111/mec.13187 25847197

[B60] Schreber J. C. D. (1775). *Die Säugthiere in Abbildungen nach der Natur mit Beschreibungen* [*Mammals Illustrated from Nature, with Descriptions*]. Erlangen: Walther. 10.11588/diglit.3115

[B61] Skinner J. D. Chimimba C. T. (2005). *The Mammals of the Southern African Subregion.* Cambridge: Cambridge University Press.

[B62] Tenaillon O. Skurnik D. Picard B. Denamur E. (2010). The population genetics of commensal *Escherichia coli*. *Nat. Rev. Microbiol*. 8 207–217. 10.1038/nrmicro2298 20157339

[B63] Terio K. A. Marker L. Munson L. (2004). Evidence for chronic stress in captive but not free-ranging cheetahs (*Acinonyx jubatus*) based on adrenal morphology and function. *J. Wildl. Dis*. 40 259–266. 10.7589/0090-3558-40.2.259 15362825

[B64] Tong S. Y. Davis J. S. Eichenberger E. Holland T. L. Fowler V. G. (2015). Staphylococcus aureus infections: epidemiology, pathophysiology, clinical manifestations, and management. *Clin. Microbiol. Rev*. 28 603–661. 10.1128/CMR.00134-14 26016486 PMC4451395

[B65] Trevelline B. K. Fontaine S. S. Hartup B. K. Kohl K. D. (2019). Conservation biology needs a microbial renaissance: a call for the consideration of host-associated microbiota in wildlife management practices. *Proc. Biol. Sci*. 286:20182448. 10.1098/rspb.2018.2448 30963956 PMC6364583

[B66] Walker A. R. Bouattour A. Camicas J. L. Estrada-Peña A. Horak I. G. Latif A. A. et al. (2000). *Ticks of Domestic Animals in Africa: A Guide to Identification of Species.* Edinburgh: Bioscience Reports.

[B67] Wasimuddin Menke S. Melzheimer J. Thalwitzer S. Heinrich S. Wachter B. et al. (2017). Gut microbiomes of free-ranging and captive Namibian cheetahs: diversity, putative functions and occurrence of potential pathogens. *Mol. Ecol*. 26 5515–5527. 10.1111/mec.14278 28782134

[B68] Weise F. J. Lemeris J. R. Munro S. J. Bowden A. Venter C. van Vuuren M. et al. (2015). Cheetahs (*Acinonyx jubatus*) running the gauntlet: an evaluation of translocations into free-range environments in Namibia. *PeerJ* 3:e1346. 10.7717/peerj.1346 26528410 PMC4627913

[B69] West A. G. Waite D. W. Deines P. Bourne D. G. Digby A. McKenzie V. J. et al. (2019). The microbiome in threatened species conservation. *Biol. Conserv.* 229:98. 10.1016/j.biocon.2018.11.016

[B70] Zhong J. Jasinskas A. Barbour A. G. (2007). Antibiotic treatment of the tick vector *Amblyomma americanum* reduced reproductive fitness. *PLoS One* 2:e405. 10.1371/journal.pone.0000405 17476327 PMC1852332

[B71] Zolnik C. P. Prill R. J. Falco R. C. Daniels T. J. Kolokotronis S. O. (2016). Microbiome changes through ontogeny of a tick pathogen vector. *Mol. Ecol*. 25 4963–4977. 10.1111/mec.13832 27588381

