# Supplementary figures and images for: A snapshot of the microbiome of blood and ticks of captive cheetahs (Acinonyx jubatus) from selected conservation facilities in South Africa
Source: Front Microbiol. 2026 Jul 20;17:1882746. doi: 10.3389/fmicb.2026.1882746 (PMC13429711; doi:10.3389/fmicb.2026.1882746)

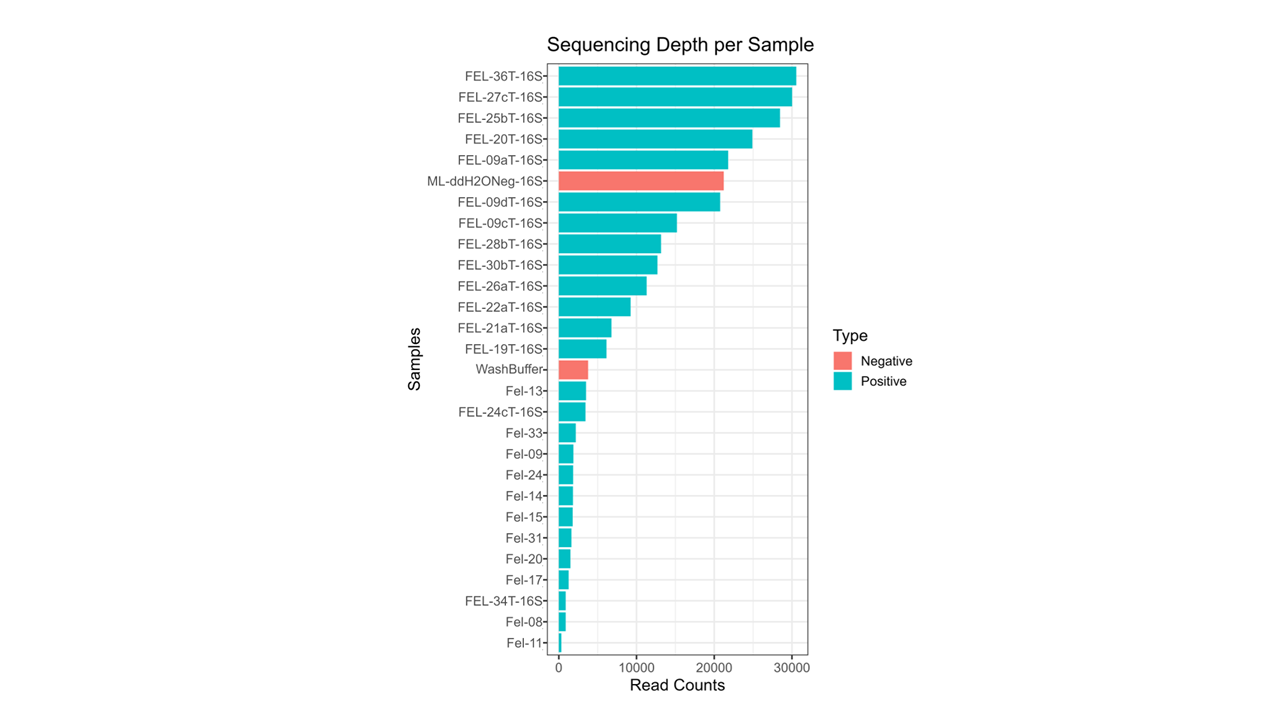

Supplement: Supplementary Figure 1 — Sequencing depth across all samples showing the total number of reads retained per sample after quality filtering. Negative controls are highlighted in red, while biological samples are shown in turquoise. [file Image_1.tif]

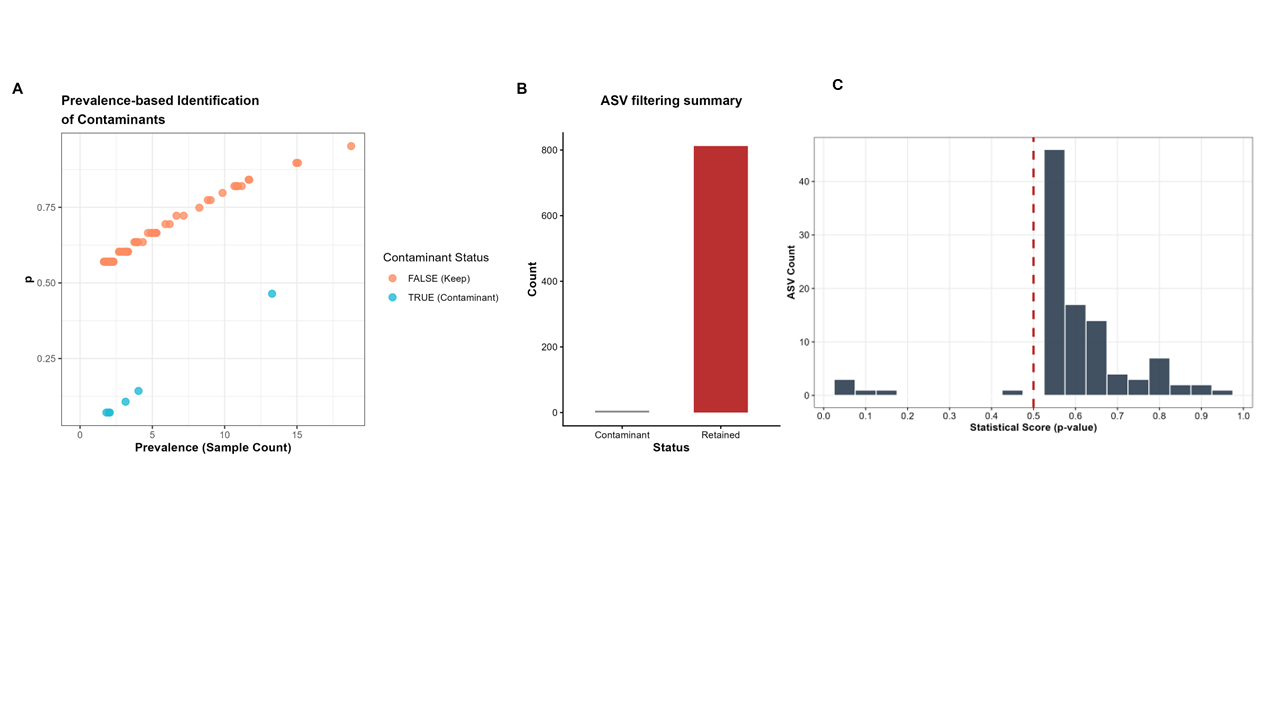

Supplement: Supplementary Figure 2 — (A) Prevalence-based decontamination analysis identified contaminant and (B) retained ASVs based on statistical probability scores. (C) Contaminant ASVs were removed prior to downstream analyses, with the red dashed line indicating the selected classification threshold (p = 0.5). [file Image_2.tif]

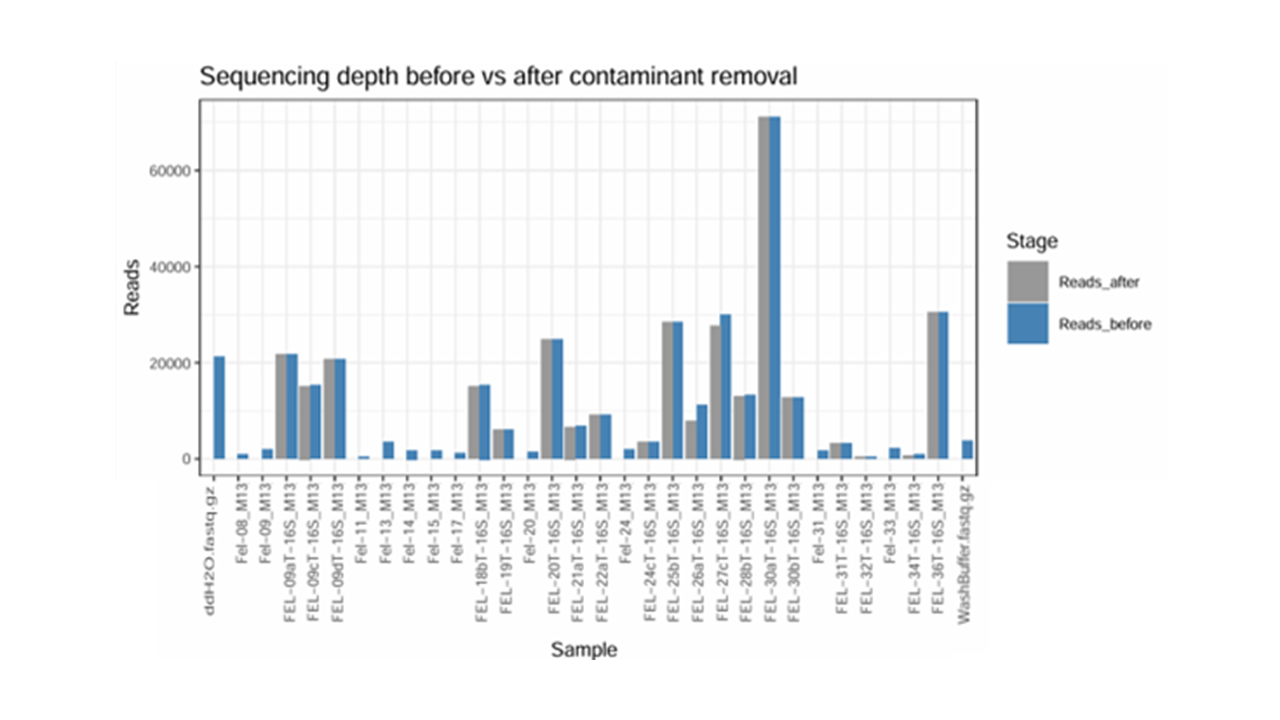

Supplement: Supplementary Figure 3 — Comparison of total read counts per sample before (blue) and after (gray) filtering for contaminants using decontam. [file Image_3.tif]

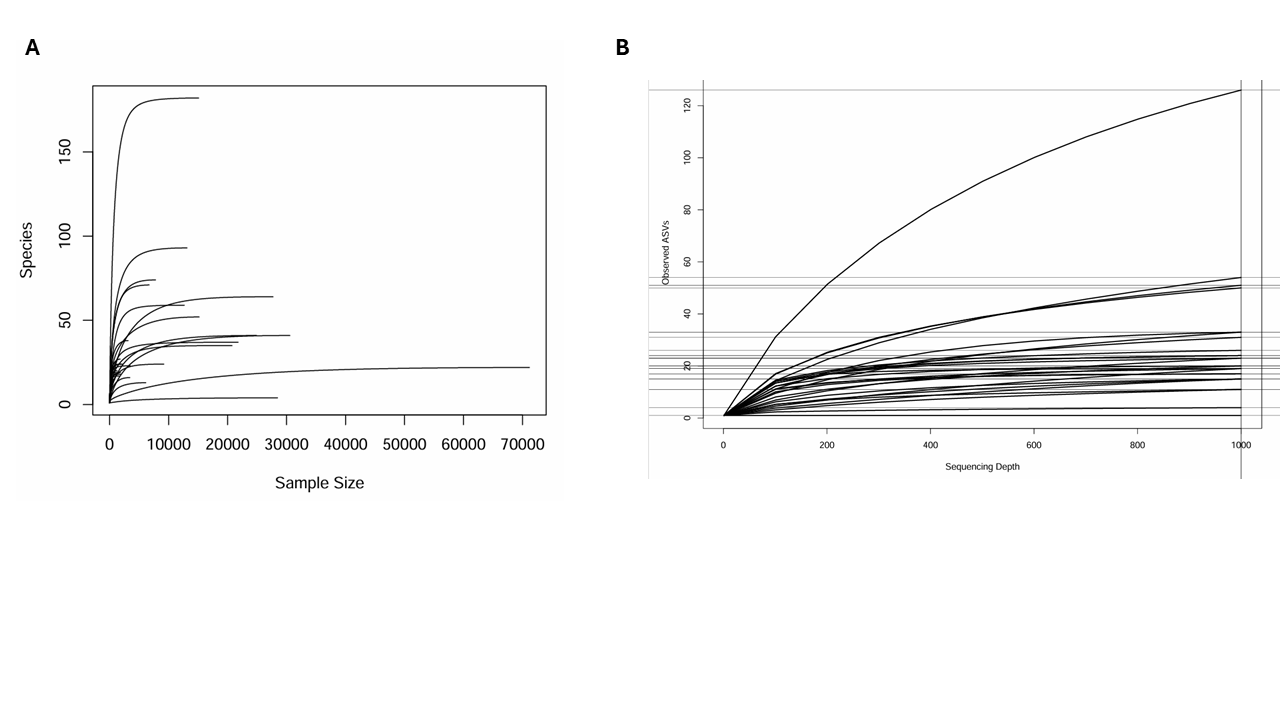

Supplement: Supplementary Figure 4 — (A) Rarefaction curves showing ASV(s) richness as a function of sequencing depth before rarefaction (A) and after rarefaction (B). [file Image_4.tif]
